# Management of intracranial melanomas in the era of precision medicine

**DOI:** 10.18632/oncotarget.19223

**Published:** 2017-07-13

**Authors:** Grace J. Young, Wenya Linda Bi, Winona W. Wu, Tanner M. Johanns, Gavin P. Dunn, Ian F. Dunn

**Affiliations:** ^1^ Department of Neurosurgery, Brigham and Women’s Hospital, Harvard Medical School, Boston, MA, USA; ^2^ Department of Cancer Biology, Dana-Farber Cancer Institute, Harvard Medical School, Boston, MA, USA; ^3^ Division of Medical Oncology, Department of Internal Medicine, Washington University School of Medicine, St. Louis, MO, USA; ^4^ Center for Human Immunology and Immunotherapy Programs, Washington University School of Medicine, St. Louis, MO, USA; ^5^ Department of Neurological Surgery, Washington University School of Medicine, St. Louis, MO, USA

**Keywords:** intracranial melanoma, BRAF inhibition, targeted therapy, immunotherapy, anti-PD1

## Abstract

Melanoma is the most lethal of skin cancers, in part because of its proclivity for rapid and distant metastasis. It is also potentially the most neurotropic cancer in terms of probability of CNS metastasis from the primary lesion. Despite surgical resection and radiotherapy, prognosis remains guarded for patients with brain metastases. Over the past five years, a new domain of personalized therapy has emerged for advanced melanoma patients with the introduction of BRAF and other MAP kinase pathway inhibitors, immunotherapy, and combinatory therapeutic strategies. By targeting critical cellular signaling pathways and unleashing the adaptive immune response against tumor antigens, a subset of melanoma patients have demonstrated remarkable responses to these treatments. Over time, acquired resistance to these modalities inexorably develops, providing new challenges to overcome. We review the rapidly evolving terrain for intracranial melanoma treatment, address likely and potential mechanisms of resistance, as well as evaluate promising future therapeutic approaches currently under clinical investigation.

## INTRODUCTION

Melanoma comprises 4% of all skin cancers but is responsible for 80% of skin cancer-related deaths [1], in part because of its proclivity for metastasis [2]. Once disseminated, median overall survival averages 6–11 months [3, 4]; overall survival is reduced to 4–5 months for metastases to the brain [5–7]. Until the last five years, treatment options for melanomas that have metastasized to the brain have been limited. Despite surgical resection and irradiation, a majority of patients experience rapid disease progression [8]. Minimal benefit has been observed with cytotoxic chemotherapy [9].

Recently, developments in molecular and immunological targeting have produced new therapeutics which have changed the landscape of melanoma treatment and prognosis. This review examines recent developments in targeted systemic treatment of advanced melanoma—including BRAF inhibitors, MEK pathway inhibitors, the blockade of the CTLA-4 and PD-1/PD-L1 immune checkpoints, and other novel anti-neoplastic agents—and highlight lessons learned from countering acquired resistance.

### Epidemiology of intracranial melanomas

Melanoma is currently the sixth most common cancer in the United States, with the highest rate of untreated fatality among cutaneous malignancies [1]. Its rate of diagnosis is increasing more quickly than any other cancer in the nation, partially because of escalated sun-seeking behavior in previous decades, greater community awareness, and higher screening rates [10, 11]. UV light is recognized as the predominant environmental risk factor and triggers distinct subgroups of melanoma with respect to chronic sun damage versus acute high-intensity exposure [12]. Melanoma is one of the most likely cancers to metastasize to the central nervous system (CNS) following lung and breast cancer [13, 14], accounting for up to 10% of patients with brain metastases [15]. Most cases of intracranial melanoma metastasize from a dermal origin, with primary intracranial melanomas a rare entity [16]. Given the paucity of systematic treatment studies in primary intracranial melanomas, we focus on the more prevalent metastatic melanoma to the brain.

Melanoma is classified by the American Joint Commission on Cancer TNM staging system, with stages I and II designating primary site melanoma, stage III denoting metastasis to one or more lymph nodes, and stage IV indicating presence of distant metastasis to other sites of the body [3]. Intracranial metastatic melanoma is classified as stage IV disease, and is usually associated with extensive lymph node involvement and visceral dissemination [17]. Half to three-quarters of patients with advanced melanoma exhibit brain metastases [18], with associated peritumoral edema, mass effect, and hemorrhage attributed as the direct cause of mortality in 94.5% of patients with intracranial tumors [7, 19]. Risk determinants for intracranial melanoma metastasis include origin from the head, neck, or mucosal regions and ulcerated or nodular lesions [7, 20]. Prognosis for those with brain metastases depends on a range of factors, including number of metastatic sites, CNS symptoms, electrocochleography (ECOG) scores, elevated serum S100 protein, elevated serum lactate dehydrogenase, advanced age, gender, and Karnofsky performance status [20–22]. Of interest, primary features may also influence prognosis, including location of primary melanoma, invasiveness, size, ulceration, and density of surrounding lymphatics and blood vessels [20].

### Conventional therapies for intracranial melanoma

The high rate of intracranial metastasis presents a considerable challenge for the management of malignant melanoma. Surgery and irradiation are commonly employed as first-line treatments, but exert only modest effects on overall survival even in the presence of local control. Conventional chemotherapy in the form of alkylating agents (dacarbazine, temozolomide) also exhibits limited efficacy.

### Surgery and/or SRS for single or oligo metastatic disease

Resection of solitary or oligo-metastases followed by whole brain radiation therapy is classically associated with significant reduction in local recurrence [23–25], although the presence of multiple lesions confers poorer prognosis [26]. Even in the setting of multiple metastases, however, a lesion causing significant mass effect and neurologic compromise or near eloquent regions of the brain may be candidate for resection.

Stereotactic radiosurgery (SRS), independently or as adjuvant therapy to the resection cavity following surgery, has shown some efficacy in controlling multiple intracranial tumors, with optimal treatment ranges between 10 and 20 Gy [27]. Indications include tumors that are smaller than 3 cm, in deep locations, without significantly associated edema, midline shift, or hydrocephalus, and relatively good prognosis. Tumor control rates with SRS alone have been reported to range from 40% to 75% [28–30], with a median survival of 8–10 months following initial diagnosis.

### WBRT as an adjunct to surgery/SRS

Adjuvant whole brain radiation therapy (WBRT) may reduce local recurrence in patients with metastatic melanoma, but does not necessarily improve overall survival [31], although higher doses of WBRT increases median survival from 13 months to 17 months [32]. In addition, the use of fractionated WBRT after resection (as opposed to focal or stereotactic radiosurgery) has been challenged due to high melanoma radioresistance rates [33] and associated complications such as radiation leukoencephalopathy, edema, and cognitive decline [34]. SRS plus WBRT may result in equivalent or better survival compared with surgery and WBRT alone, although the survival advantage remains modest [23, 35].

### Palliative WBRT

Of note, primary administration of WBRT (e.g., 30 Gy administered in 10 fractions) may be provided to patients who are not resection candidates due to location or number of metastases as a palliative measure. Median survival in these cohorts has been reported to increase from 2.1 months to 3.6- 4.8 months [7, 36–38].

### Conventional chemotherapy

Conventional chemotherapies have been utilized for patients with extensive disease burden with limited success. Dacarbazine was the first FDA-approved chemotherapy for melanoma, but conferred an overall response rate of only 10–20%, with less than 5% complete response [39–41] and mean survival of less than 8 months [42, 43]. Lack of efficacy has been attributed to an inability to transverse the blood-brain barrier (BBB) and the activity of efflux pumps such as breast cancer resistance protein and P-glycoprotein [44]. Fotemustine, a nitrosurea capable of crossing the BBB [45], subsequently demonstrated marginally improved median progression-free survival (PFS) by 1–2 months relative to dacarbazine, with response rates of 5–6% [46, 47].

Temozolamide is a well-tolerated systemic therapy that does not significantly improve objective response rate and progression free survival as a single agent, but may be promising in combination therapy with other drugs [48]. Recent phase 1/2 preliminary data for decitabine—a DNA methyltransferase inhibitor attenuating CpG methylation in metastatic melanoma—in combination with temozolamide indicated an overall response rate of 18% and a median overall survival of 12.4 months [49].

Prophylactic chemoprevention has shown reasonable success in several studies for cancers other than melanoma [50]. In the case of melanoma, as metastases employ integrin signaling to grow along blood vessels, future approaches may opt to administer integrin inhibiting agents (e.g., cilengitide) during early disease progression to delay intracranial colonization [51]. In practice, however, cancer chemoprevention is seldom utilized in the clinical setting [52].

### Limits of conventional therapies

To date, conventional treatment options have provided limited strategies to target metastatic melanoma. Surgical resection and irradiation are often not feasible in many melanoma cases due to the multiplicity of intracranial metastases and their relative radioresistance. With emerging understanding of tumor heterogeneity across space and time [53], melanoma brain metastases also likely represent diverse entities with varying sensitivities to treatment and ready acquisition of resistance. Recurrent disease and progressive metastases further thwart the effectiveness of systemic therapies. In this backdrop, the advent of targeted therapies for metastatic melanoma in the past 5 years has offered renewed promise.

### Mechanisms of melanoma metastasis to the brain

The identification of actionable mechanisms of metastasis in any cancer type remains a “holy grail” of cancer research, as disease dissemination is the main driver of mortality. Although much remains to be learned in our understanding of how melanoma cells metastasize to the brain, a number of mechanisms are likely to contribute to this process.

### Cerebral chemotaxis

Melanoma metastasis is facilitated by the well-perfused dermal capillary and lymphatic network at its site of origin [54]. A combination of autocrine and paracrine factors influence melanoma chemotaxis and survival in the brain [55–57]. Melanoma cells express low-affinity neurotrophin receptors p75NTR and TrKc, which are positively regulated by nerve growth factor (NGF) and neutrophin-3 (NT-3), leading to enhanced production of extracellular matrix (ECM) degradative enzymes such as heparanase and culminating in the breakdown of the blood-brain barrier (BBB). Chemokines and their receptors are also implicated in preferential homing of melanoma cells to target sites, including CXC chemokine receptor 4 (CXCR-4) and C-C chemokine receptor 7 (CCR-7) [58]. Recently, expression of the protein PLEKHA5 has been demonstrated to be higher in cerebrotropic metastases compared to systemic melanomas, suggesting an additional biomolecular target for future study [59].

### Cytokine signaling

Paracrine cytokine signaling by activated astrocytes and microglia may further stimulate tumor development. Vascular endothelial growth factor A (VEGF-A) and IL-6, in particular, promote accelerated growth and infiltration of melanoma cell lines *in vitro* with accompanying co-optation of peritumoral vessels and increased vascular permeability [60]. VEGF expression in melanoma metastases can be upregulated by loss of suppressor of cytokine signaling-1 and ensuing activation of signal transducer and activator of transcription 3 (STAT3) [61]. Other upregulated genes in advanced intracranial melanoma include angiopoietin-like protein 4, cyclooxygenase-2 (COX-2), and matrix-metalloproteinase-1 (MMP-1) [55].

### Immune evasion

After seeding the CNS, melanoma metastases are subject to the unique immunologic environment provided by the brain. Interestingly, murine and human tumor cells have been shown to lose normal expression of the tumor suppressor PTEN after metastasis to the brain, but not to other organs, which is then reinstated after leaving the CNS environment. The mechanism of plasticity is postulated to be produced by astrocyte-dependent, epigenetically regulated protein downregulation, where astrocyte-derived exosomes arbitrate an intercellular exchange of PTEN-targeting microRNAs to metastatic tumor cells, while depletion of these microRNAS or blockade of astrocyte exosome secretion effectively “rescues” PTEN expression and suppresses brain metastasis [62].

In contrast to the historic view of the CNS as an immunologically privileged compartment, bounded by the blood-brain barrier, research increasingly reveals that CNS antigens are in fact accessible to peripheral lymphoid tissues and able to mount significant anti-tumoral immunity [63–65]. Furthermore, the number of intratumoral lymphocytes positively correlates with increased patient survival; metastatic melanoma patients with brisk lymphocyte activity demonstrated 1.5–3 times longer overall survival compared to those without, although this was not a brain-specific finding [66, 67]. Recently, a functional lymphatic network abutting the dural sinuses was identified that provides an anatomic conduit for immune cell influx and efflux to the CNS [68, 69]. Taken together, these data suggest that immune potentiation may be a potent strategy for targeting CNS metastases.

### Molecularly targeted therapies for melanoma

In 2011, the treatment of metastatic melanomas was revolutionized by FDA approval of two new agents: vemurafenib (Zelboraf^®^; Genentech, San Francisco, California, USA) and ipilimumab (Yervoy^™^; Merck, Kenilworth, New Jersey, USA). Both address critical and independent pathways of advanced melanoma growth: the mitogen-activated protein (MAP) kinase signaling pathway for vemurafenib [70, 71] and the CTLA-4 signaling pathway for ipillimumab [72, 73]. These new therapies heralded a paradigm shift by providing rational targeted therapies for a disease with a poor prior history of treatment (Table 1).

**Table 1:** FDA-approved molecular and immune targeted therapies for melanoma

Drug Name	Mechanism	Target tumor population	FDA Approval Date	FDA Recommended Administration Protocol	Response Rate	Side Effects	Resistance Onset	Treatment options after onset of resistance
**Molecular Targets**								
Vemurafenib (Zelboraf^®^)	BRAF V600E/K inhibitor	*BRAF* V600E/K mutant	August 2011	960 mg 2x daily	30–39%, in intracranial patients^86, 88^	Novel primary malignancies, tumor promotion in *BRAF* wild-type patients, hypersensitivity reactions, dermatologic reactions, QT prolongation, hepatotoxicity, photosensitivity, opthamologic reactions^94–96^	7 months, frequent^107–111^	Anti-BRAF/MEK co-therapy, dual Ras-Mek-Erk/PI3K-PKB inhibition, ERKi, intermittent dosing, combination of BRAFi with immunotherapies (e.g., anti-CTLA, anti-PD1, anti-PD-L1)^109–117, 119, 123–126^
Dabrafenib (Tafinlar^®^)	BRAF V600E/K inhibitor	*BRAF* V600E/K mutant	May 2013	150 mg 2x daily	31–52%, in intracranial patients^88–89^	Novel prima ry malignancies, tumor promotion in BRAF wild-type patients, hemorrhage, venous thromboembolism, cardiomyopathy, ocular toxicities, febrile reactions, skin toxicity, hyperglycemia, glucose-6-phosphate dehydrogenase deficiency^94–96^		
Trametinib (Mekinist^™^)	MEK inhibitor	*BRAF* V600E/K mutant	January 2014	2 mg 1x daily	54%^100^	Novel primary malignancies, tumor promotion in BRAF wild-type patients, hemorrhage, venous thromboembolism, cardiomyopathy, ocular toxicities, interstitial lung disease, skin toxicity, febrile reactions, hyperglycemia^115^		
Cobimetinib (Cotellic^®^)	MEK inhibitor	*BRAF* V600E/K mutant	November 2015	60 mg 1x daily for the first 21 days of each 28-day cycle until disease progression or unacceptable toxicity, in combination with vemurafenib	68%^102^	Central serous retinopathy, gastrointestinal events, photosensitivity, elevated aminotransferase levels, and an increased creatine kinase level^102^	9.9 months, frequent^102^	
**Immunotherapy**								
Ipilimumab (Yervoy^®^)	CTLA-4 inhibitor	Unrestricted	March 2011	3 mg/kg administered intravenously every 3 weeks	15–30%, in intracranial patients^145^	Immune-mediated enterocolitis, immune-mediated hepatitis, immune-mediated dermatitis, immune-mediated neuropathies, immune-mediated endocrinopathies^148, 136^	> 1 year, rare^61^	Other immunotherapies, histone-deacetylase inhibition, indomethacin, ACT, combination SRS and immunotherapy^185–187^
Nivolumab (Opdivo^®^)	PD-1 inhibitor	Unrestricted	December 2014	3 mg/kg administered intravenously every 2 weeks	28%^155^	Immune-mediated pneumonitis, immune-mediated colitis, immune-mediated hepatitis, immune-mediated nephritis and renal dysfunction, immune-mediated hypothyroidism and hyperthyroidism^155^		
Pembrolizumab (Keytruda^®^)	PD-1 inhibitor	Unrestricted	September 2014	2 mg/kg administered intravenously every 3 weeks	37–38%^156^	Immune-mediated pneumonitis, immune-mediated colitis, immune-mediated hepatitis, immune-mediated hypophysitis, immune-mediated nephritis and renal dysfunction, immune-mediated hyperthyroidism and hypothyroidism^156^		

### Role of BRAF mutations in melanoma malignancy

Mutations in *BRAF* are the most common targetable genetic variant in advanced melanoma [74]. *BRAF* mutations are found in 40–60% of melanomas, with a valine to glutamic acid substitution at residue 600 (V600E) as the most common variant (75–80%), followed by substitution to lysine (V600K, 17–22%) and arginine (V600R, 3–4%) [75, 76]. Differences in *BRAF* genetic variants (V600E vs. other mutations) appear to be dependent on patient demographics and primary disease site, with non-V600E tumors occurring more frequently in men, older patients, and truncal lesions [77, 78]. Tumors with non-V600E mutations also have a shorter disease-free interval from diagnosis of primary melanoma to first distant metastasis compared to their V600E counterparts [78]. Prognosis is less favorable for non-V600E mutations as efficacy of targeted therapy is more limited [78].

Raf exists in three mammalian isoforms: ARAF, BRAF, and CRAF (also known as Raf-1), of which the latter two are proto-oncogenic. Mutant BRAF triggers constitutive activation of the MAPK/ERK pathway and is typically mutually exclusive of activating *RAS* mutations in tumors [74, 79]. Other BRAF-mediated mechanisms in melanoma include UV activation of BRAF-driven tumorigenesis through single nucleotide mutations in *TP53* [80] and copper-promoted MEK1 phosphorylation of ERK [81]. Co-occurrence with telomerase reverse transcriptase (TERT) promoter mutations (found in 38% of melanomas) was observed to be more commonly associated with high-risk clinicopathologic characteristics [82], suggesting potential interplay of *TERT* promoter and *BRAF* mutations in melanoma evolution.

### BRAF inhibitors for invasive melanoma

Vemurafenib is an inhibitor of the BRAF serine threonine kinase mutant and blocks its constitutive activation of the MAPK mitogenic signaling pathway. A phase 2, multi-center study of vemurafenib in 132 previously treated *BRAF*^V600^ mutant metastatic melanoma patients showed a response rate of 53%, a median duration of response of 6.7 months, and a median overall survival of 15.9 months [83]. Interim analysis during a phase 3 randomization of 675 previously untreated *BRAF*^V600E^ mutant metastatic melanoma patients to vemurafenib or dacarbazine revealed significant benefit for the vemurafenib cohort; 80% of patients showed a reduction in tumor size, with over half showing a reduction of > 30% [71, 84]. Median overall survival (OS) and PFS both increased by 4–5 months in the vemurafenib arm.

Most initial clinical trials excluded patients with metastases to the CNS, unless such metastases had been definitively treated with no signs of progression or need for glucocorticoid therapy for 3 months or more prior to initiation of treatment [71]. Nevertheless, isolated reports emerged suggesting positive effects of vemurafenib in controlling growth and reducing mass effect for intracranial melanoma metastases with the BRAF^V600E^ mutation [85]. A subsequent pilot study found that twice daily administration of vemurafenib at 960 mg was associated with >30% intracranial tumor regression in 37% of patients and intracranial partial response in 16% [86]. In previously untreated patients, overall intracranial response was 39% in tumors with the *BRAF*^V600E^ mutation and 7% in tumors with the *BRAF*^V600K^ mutation. In *BRAF*^V600^-mutant melanoma patients who had received previous treatment for unresectable brain metastases, overall intracranial response was 31% and 22% for V600E and V600K mutant tumors, respectively [86]. Median OS and PFS were 5.3 months and 3.9 months; 92% of patients were discontinued from the study within 4 months due to disease progression. As the brain is a common site of treatment failure (20%–25%) in patients without known brain metastases who are treated with vemurafenib, this suggests that vemurafenib monotherapy may be insufficient for intracranial metastatic melanoma [87]. A phase 2 study evaluating vemurafenib in a larger group of *BRAF*-mutant patients with active brain metastases is currently underway (NCT01378975).

In recognition of the potential of BRAF inhibition, dabrafenib (Tafinlar®; Novartis, Mission Viejo, California, USA) has also been FDA-approved. Drug efficacy was demonstrated in patients with active brain metastases, something that was not originally evaluated for vemurafenib [88]. In a phase 2 trial of *BRAF*^V600E^ or *BRAF*^V600K^ mutant melanoma metastases to the brain, 39% of previously untreated *BRAF*^V600E^ patients demonstrated a response while 31% of *BRAF*^V600E^ patients with disease progression following prior surgery or radiation therapy also demonstrated a response. Median OS was 31–33 weeks for patients with *BRAF*^V600E^ metastases, as compared to 16–22 weeks for those with *BRAF*^V600K^ mutant tumors; PFS was 16 weeks for *BRAF*^V600E^ patients and 8–16 weeks for *BRAF*^V600K^ patients, depending on prior treatment status [88]. A phase 3 randomized trial for dabrafenib in 250 patients with V600E-mutant stage III or IV melanoma showed a 50% response rate and an improved PFS over dacarbazine (5.1 vs. 2.7 months) [89].

### Blood barrier efficacy for BRAF inhibitors

Although the mechanism of blood-brain transport is unclear, the response and improved survival curves in patients treated with BRAF inhibitors suggest some level of inhibitor penetrance in patients with active cranial melanoma metastases [86, 90]. It serves to note that a range of factors may play a role in penetrance efficacy, including the number and size of brain metastases, the presence of co-morbidities, neurological complications, and tumor mutation status [73, 91].

In animal studies, BRAF inhibitors have been shown to be substrates for barrier efflux pumps, with increases up to 10-fold in brain distribution for P-glycoprotein and breast cancer resistance protein-1 knockouts [92]. Greater CNS penetration is observed for dabrafenib than for vemurafenib under similar dosing schedules [93]. Development of delivery mechanisms inhibiting the activity of efflux transporters may further increase the therapeutic efficacy of BRAF inhibition in intracranial disease.

### Side effects of BRAF inhibition

Long-term administration of BRAF inhibitors has been observed to associate with a range of skin changes, febrile reactions, arthralgia, headache, venous thromboembolism, cardiomyopathy, and hyperglycemia [94]. More concerning, secondary malignancies including squamous cell carcinomas, secondary melanomas, and recurrence of pre-existing malignancies have also been reported [95, 96]. These are predominantly attributed to MAPK pathway activation and increased Ras activity in BRAF wild-type cells exposed to BRAF inhibitors [97], adding a cautionary note to avoid BRAF inhibitor therapy in melanoma patients without mutant *BRAF* status.

### MEK inhibition

**Figure 1:**
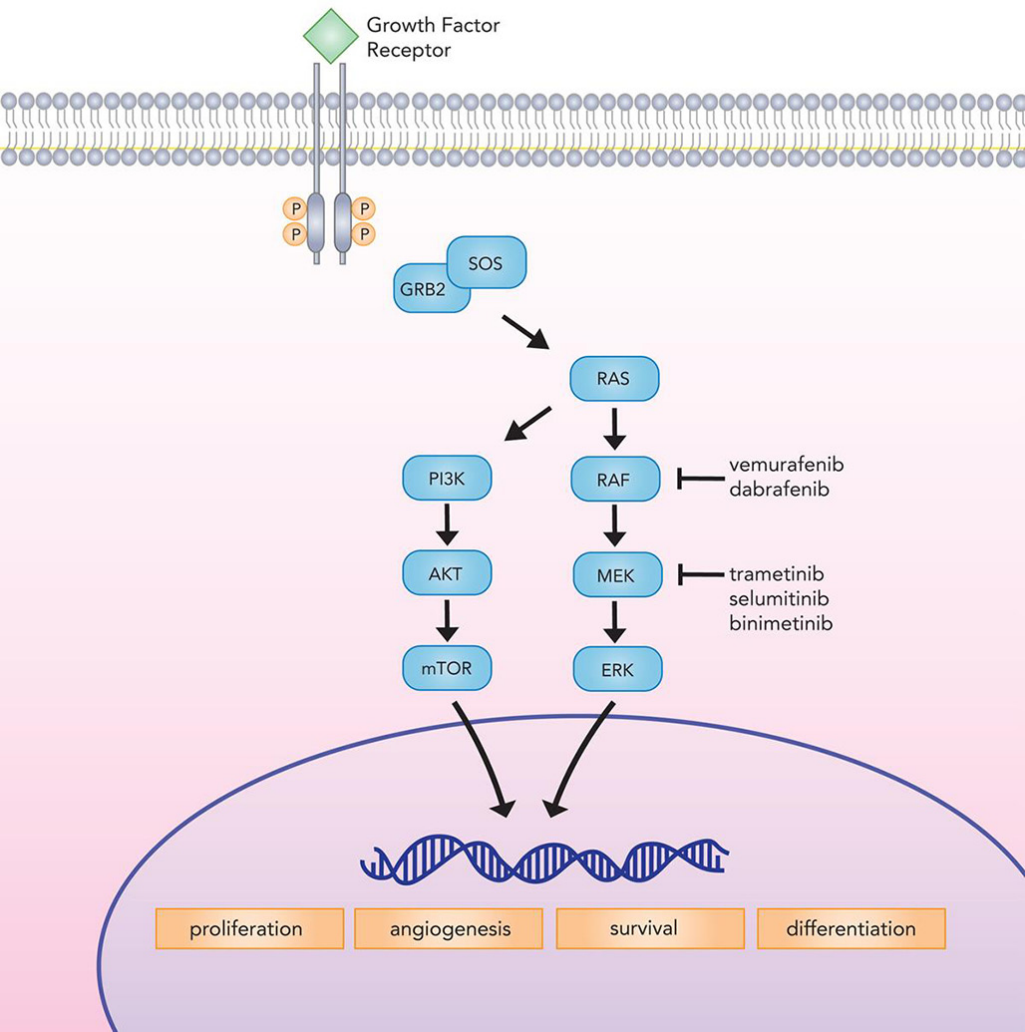
Canonical MAP kinase signaling mediates critical cellular processes implicated in proliferation, differentiation, survival, and angiogenesis BRAF inhibitors (vemurafenib and dabrafenib) and MEK inhibitors (trametinib, selumitinib, and binimetinib) target the MAP kinase pathway to check tumor growth.

The presence of *BRAF* mutation is also associated with enhanced and selective sensitivity to MEK inhibition in melanoma cells [98]. Concomitant with the development of BRAF inhibitors, several MEK inhibitors—including trametinib, selumitinib, and binimetinib [99]—have emerged in recent years, adding to the existing pharmacologic armamentarium. (Figure 1)

Trametinib (Mekinist™ Novartis, Mission Viejo, California, USA), a MEK 1/2 inhibitor, has shown therapeutic promise as either a monotherapy for *BRAF*-mutant melanoma or in combination with dabrafenib. A phase 3 trial comparing trametinib to dacarbazine or paclitaxel in metastatic *BRAF*^V600^-mutant melanoma patients demonstrated significant improvement in median PFS and a 6-month survival rate of 81% despite a 47% crossover rate from the chemotherapy group [100]. Of note, only 4% of the patients receiving trametinib harbored a known brain metastasis at study enrollment. Combination therapy with dabrafenib or cobimetinib results in markedly improved response, with median PFS in phase 3 studies ranging from 9.3–9.9 months for combination treatment versus 8.8 months for dabrafenib alone [101] and 6.2 months for cobimetinib alone [102]. Although these studies did not include patients with brain lesions, a number of phase 2 studies (COMBI-MB, co-BRIM3, NCT01978236) are currently investigating the effect of BRAF/MEK inhibitory therapy on active brain metastasis.

Cobimetinib (Cotellic^®^; Genentech, San Francisco, California, USA), another MEK inhibitor, appears to have a favorable profile when combined with vemurafenib in patients with advanced *BRAF*-mutant melanoma. Progression-free survival during a multi-center phase 3 study was reported to reach 9.9 months in the combination group (vemurafenib + cobimetinib) and only 6.2 months in the control group (vemurafenib alone) [102]. Response rate in the combination arm was 68% versus 45% (control), with respective complete response rates of 10% and 4%. Vemurafenib and cobimetinib administered in combination was associated with some increase in toxicity, with rash, diarrhea, photosensitivity, and hepatic-enzyme abnormalities as adverse events. Incidence of secondary cutaneous cancers decreased with combination treatment. Patients with brain metastases were allowed to enroll so long as the lesion had been inactive for > 3 weeks.

As with BRAF inhibition, the CNS penetrance of MEK inhibition is unclear, although animal models suggest restriction by barrier efflux pumps [103]. Given that clinical trials have not included a substantial number of patients with brain metastases, future directions may include the exclusive testing of a CNS disease cohort to better evaluate blood brain barrier efficacy.

### Acquired resistance to BRAF and MEK inhibition

**Figure 2:**
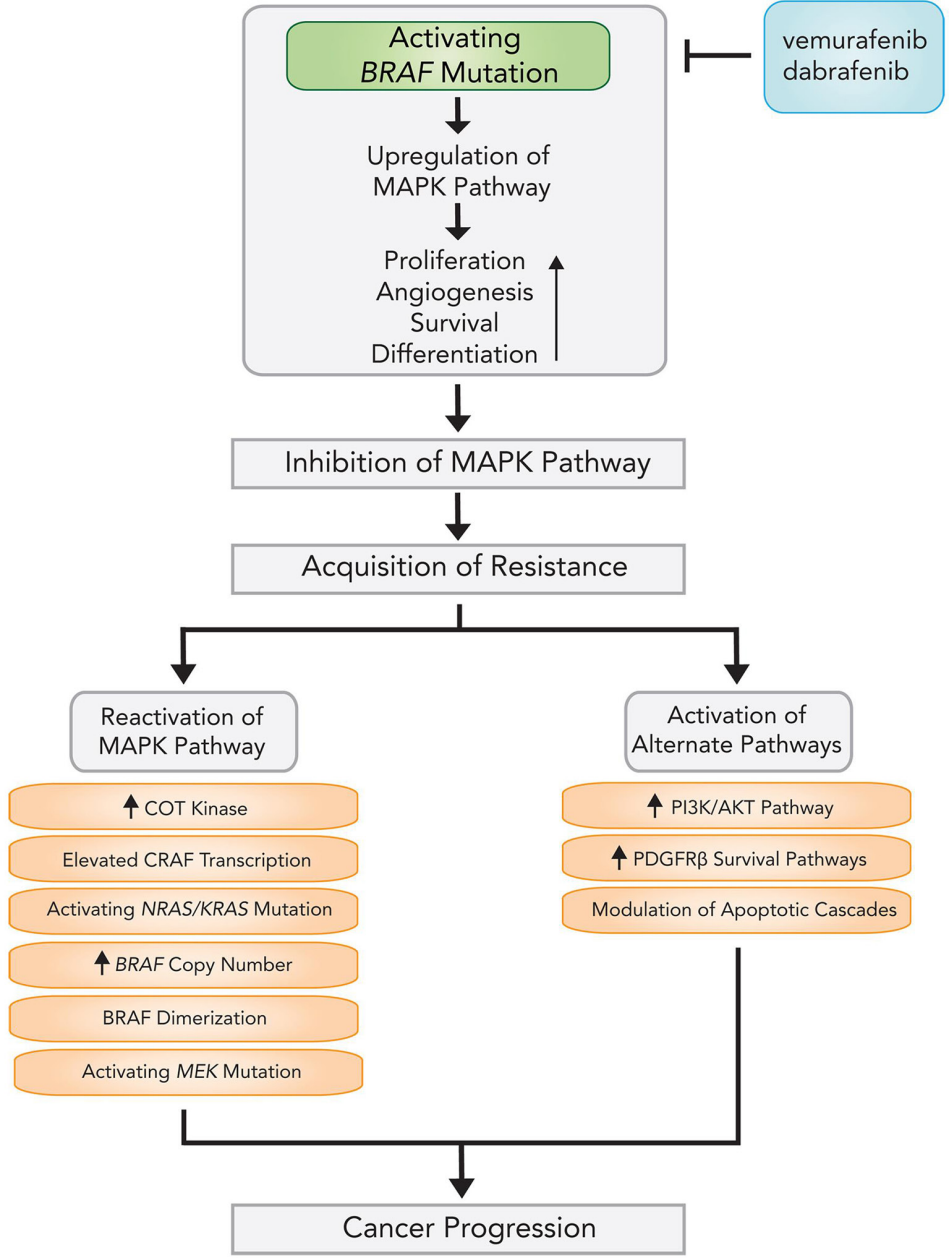
Mechanisms and pathways of acquired resistance following BRAF inhibitor therapy

One major drawback to BRAF inhibition is a rapid onset of resistance, with average time to acquired resistance to kinase inhibition being less than 7 months [104]. A majority of currently recognized resistance mechanisms culminate in Ras-Raf-MEK-ERK pathway reactivation (Figure 2), mutation of downstream *MEK1* (also known as *MAP2K1*) kinase [105], activating mutations of neuroblastoma viral RAS oncogene homolog (*NRAS*) [106] and *KRAS* [107], and dimerization of alternatively spliced BRAF [108]. *BRAF*^V600E^ copy number amplification (observed in 8–20% of resistant melanoma samples) [107, 109] and elevated *CRAF* transcription [110] can also re-instigate ERK phosphorylation. Activating *MEK* mutations are found in less than 15% of cases, with only a subset capable of driving resistance to BRAF inhibitors [109].

ERK-independent pathways are also implicated, including the PI3K-protein kinase B (PKB)-mechanistic target of rapamycin (mTOR) pathway and the caspase-dependent apoptotic cascade. These pathways converge at formation of eukaryotic translation initiation complex 4F (eIF4F), which confers resistance to inhibition of BRAF [111]. Platelet-derived growth factor receptor β (PDGFRβ) initiation of survival pathways auxiliary to MAPK [106] and EGFR upregulation have also been observed after development of resistance [112]. In addition, BRAF inhibitors may be subject to extrusion via efflux pumps expressed by blood-brain-barrier epitheliocytes [92], allowing increased transporter expression to provide a supplementary route for intracranial BRAF inhibitor resistance.

Multiple mechanisms of resistance can develop simultaneously. In an analysis of 100 resistant melanoma samples from 44 patients, 70% of tumors demonstrated a MAPK alteration and 22% demonstrated a PI3K-PKB alteration, with 20% of patients harboring at least two distinct mechanisms of resistance [107]. Awareness of such intratumoral heterogeneity may help steer strategies for countering acquired resistance to inhibitor therapy [109].

### Overcoming BRAF and MEK inhibitor resistance

One strategy for countering resistance is to concurrently target multiple oncogenic pathway members. A phase 2 study comparing combination therapy with dabrafenib and trametinib to dabrafenib monotherapy observed an unprecedented overall survival rate of 23.8 months in the co-therapy group [113]. Phase 3 comparison of dabrafenib/trametinib versus dabrafenib alone corroborated a slightly improved PFS with combination treatment (9.3 vs. 8.8 months) [114]. Notably, combination therapy was associated with a lower rate of cutaneous squamous cell carcinoma and hyperkeratoses compared to monotherapy inhibition, although higher rates of severe grade pyrexia were observed [114]. Patients with CNS dissemination were not included in these studies; the effect of BRAF/MEK inhibitor therapy on brain metastases is presently being investigated in phase 2 trials (NCT02039947, NCT02230306).

In comparison, sequential administration of a single-agent MEK inhibitor following acquired dabrafenib or vemurafenib resistance has proven largely unfruitful. A phase 2 study of trametinib in BRAF inhibitor-naïve patients versus those previously treated with dabrafenib or vemurafenib demonstrated response rates only in the drug-naïve arm (25% vs. 0%; patients with active brain metastases excluded) [115]. This likely results from multiple factors, including tumor heterogeneity among patients, cross-resistance between BRAF and MEK, and the activation of alternative signaling pathways [107, 109, 116]. Interestingly, a phase 1/2 study evaluating dabrafenib/trametinib co-therapy following dabrafenib resistance showed response rates of 13% (enrollment included patients with > 3 month history of stable brain involvement); an additional 44% experienced disease stabilization of ≥ 8 weeks [117]. Patients receiving dabrafenib monotherapy for greater than 6 months before progression and subsequent co-therapy had a greater PFS compared to those with early progression after less than 6 months of monotherapy (3.9 vs. 1.8 months). This indicates a potential role for combination therapy after acquired resistance, although effect sizes appear to be modest.

Inhibition of ERK, further down the MAPK cascade, may offer additional benefit. ERK inhibition *in vitro* is more effective than MEK inhibition in the background of *Ras* mutations, *MEK* mutations, and *BRAF* amplication [118, 119]. As ERK belongs to the same pathway as BRAF, however, inhibition may have limited efficacy following resistance and may actually promote relief of ERK-driven negative feedback on Ras signaling [120].

Emerging evidence indicates that resistance to BRAF inhibitors confers cross-resistance to MEK inhibitors, and that targeting a single pathway may not be enough for long-term disease control [116]. In addition, suppression of either Raf-MEK-ERK or PI3K-PKB signaling leads to greater activation of the alternate pathway, suggesting that inhibition of both pathways is necessary for response durability [121]. Preliminary clinical trials assessing dual pathway inhibition are currently underway [122].

An empirical model has also proposed a role for “drug holidays” in countering acquired resistance. A proportion of cells expressing mutant or amplified *BRAF* become dependent on BRAF inhibition for a selective advantage, and cessation of drug administration leads to tumor regression; a staggered regimen has been shown to delay onset of resistance in both mice and human xenograft cell lines [123]. Subsequent studies investigating aberrantly spliced BRAF showed greater rates of proliferation in the presence of PLX4720 (a next-generation BRAF inhibitor) and increased sensitivity to BRAF inhibition after a period of cessation [124]. Reversal of acquired EGFR expression has also been observed in resistant cell lines after interruption of therapy [112]. Preliminary anecdotal evidence suggests that sensitivity can be re-acquired following treatment intermission [125, 126]. A phase 2 trial is underway to evaluate long-term efficacy of intermittent dosing (2 weeks on, 2 weeks off) of BRAF inhibitor LGX818 in patients with BRAF mutant metastatic melanoma (NCT01894672).

Additional strategies to overcome resistance include combination of MAPK pathway inhibitors with immunotherapies. This arrangement is currently being evaluated in a phase 1 study with BRAF^V600^ mutant advanced melanomas [127]. Other strategies combining targeted therapies and immunotherapies with anti-programmed cell death protein 1 (PD-1) are also in development, as are studies of programmed death ligand 1 (PD-L1) checkpoint inhibitors [128]. Therapies combining BRAF inhibitor PLX4720 and anti-CCL2 or agonistic anti-CD137 antibodies have also shown significant antitumor activity in murine transplant and tumorigenesis models [129, 130].

### Immunotherapy for metastatic melanoma

**Figure 3:**
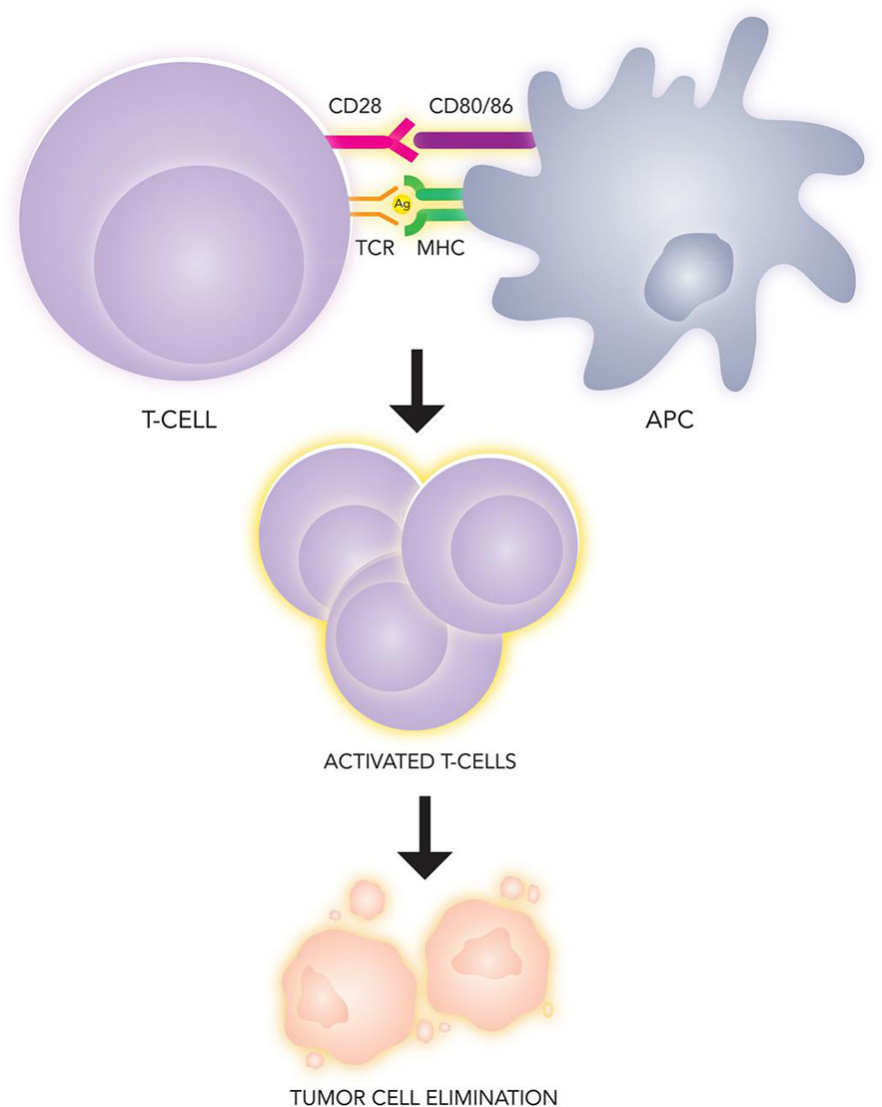
Immunologic recognition and elimination of tumors Tumor antigen is presented by an antigen presenting cell (APC) on the major histocompatibility complex (MHC), which serves as the ligand for the T-cell receptor (TCR). A second costimulatory signal (CD80/86) binding to CD28 on the lymphocyte is necessary for T-cell effector phase activation.

**Figure 4:**
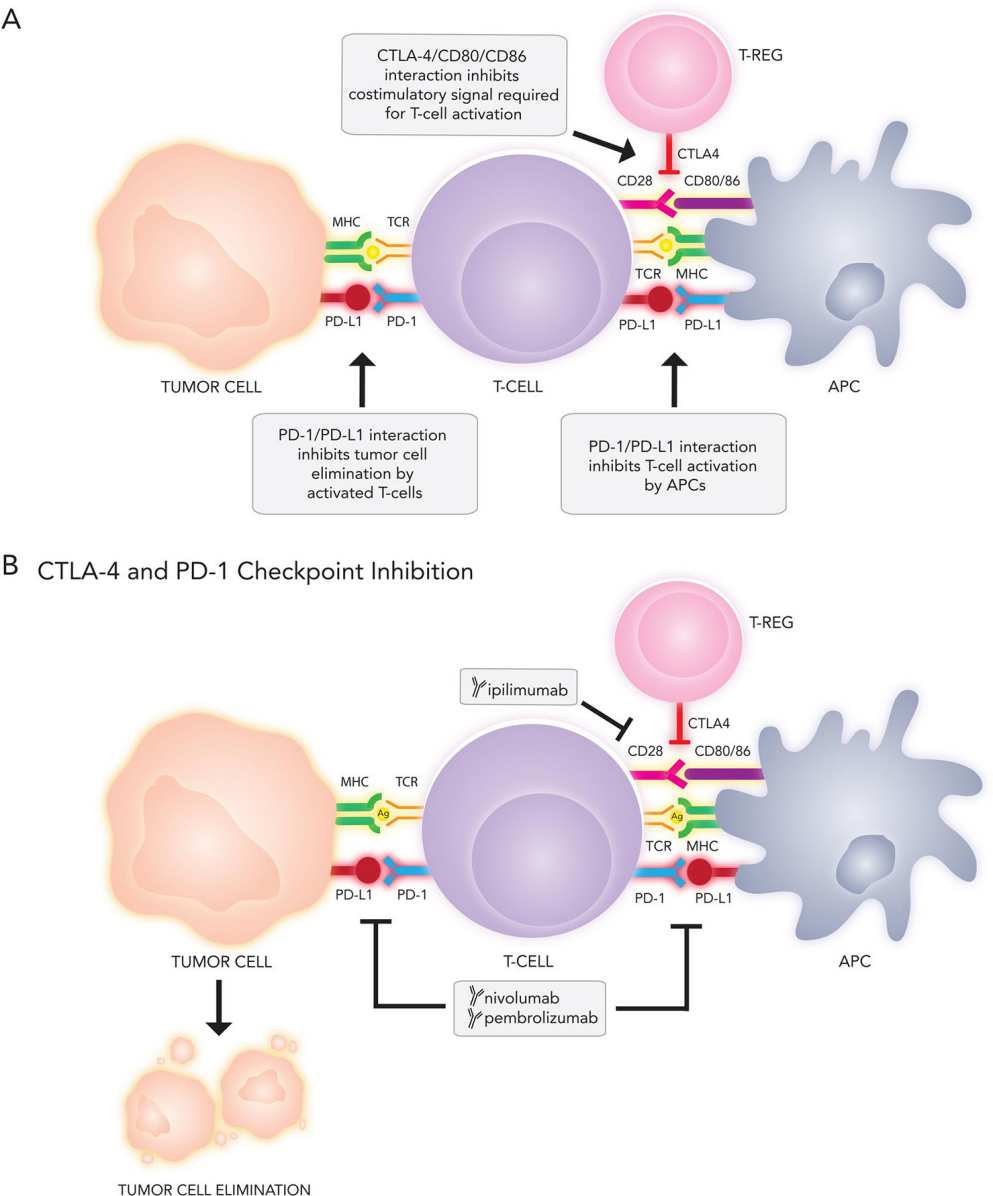
(**A**) Immune targets and (**B**) current immunotherapies under investigation for metastatic melanoma. APC, antigen presenting cell; CTLA-4, cytotoxic T-lymphocyte associated protein 4; MHC, major histocompatibility complex; PD-1, programmed cell death 1; PD-L1, programmed cell death ligand 1; TCR, T-cell receptor; T-reg, regulatory T-cell

Immunotherapy represents one of the most dramatic advances in cancer therapy over the last 5 years, and the treatment of melanoma has unquestionably been at the forefront of this revolution. An integrated immune response involving both innate and adaptive compartments is responsible for countering malignancy by activating T-cells into an effector state in a two stage process [64] (Figure 3). First, naïve T cells must recognize tumor-specific antigens presented by the MHC molecules on antigen presenting cells in secondary lymphoid tissue. The securement of antigen and its presentation to T cells is the definitive step in the afferent pathway of the immune response. Second, a costimulatory signal completes the activation, which is negatively regulated by several inhibitory molecules expressed on T cells, antigen presenting cells (APC), and tumor cells [131]. These antagonist signals are known as immune checkpoints and include cytotoxic T-lymphocyte antigen-4 (CTLA-4), an inhibitory receptor on lymphocytes which acts in the early priming phase of the immune response. The subsequent efferent pathway of adaptive immunity involves activated T cells homing to the tumor environment, where the PD-1/PD-L1 receptor-ligand pair acts to dampen peripheral immune activity (Figure 4). Melanomas are capable of inciting robust immune responses, making immunotherapy options particularly relevant. Despite instigating a heavy T-cell response, however, a proportion of the total CD8+ T cells in melanoma patients are functionally unresponsive to melanoma antigens [132]. Melanoma somehow appears to be capable of rendering T-cells anergic *in vivo*, either through CTLA-4, PD-1, mutational burden, or other molecular means. The targeting of immune checkpoints thus provides an empiric methodology for increasing the endogenous anti-tumor response.

Recent experience with immune checkpoint inhibitors has resulted in impressively durable responses in a subset of patients, in comparison to higher response rates achieved with MAPK pathway inhibition that are often less durable [133]. This may potentially be explained by the plasticity of the immune response, allowing for adaptation to an evolving tumor, as opposed to molecularly targeted drugs that can be foiled by newly acquired mutations [134, 135].

Of note, although many of the novel immunotherapeutic agents cannot cross the blood-brain barrier, CD8+ cytotoxic T cells appear to be able to travel to the brain and mount a significant anti-tumor immune response [136, 137]. Supporting this evidence, recent findings have suggested an extensive dural lymphatic system that is capable of carrying both CSF and immune cells into the murine brain; further support for this system in humans remains to be demonstrated [68].

### CTLA-4 inhibition

In cases of malignancy, as first shown by James Allison [138], CTLA-4 operates mechanistically by outcompeting the T-cell costimulatory molecule, CD28, for binding to the antigen-presenting cell expressed activation ligands, CD80/CD86 (Figure 4A), which impairs cytolytic T-cell lymphocyte activation and clonal expansion [139], inhibition of helper T-cell activity, and enhancement of the immunosuppressive activity of regulatory T-cell units [140]. Inhibition of CTLA-4 leads to significantly increased immune recognition of tumor cells [141]. In patients with advanced melanoma, CTLA-4 inhibition has been observed to produce durable response rates in approximately a quarter of treated patients [142, 143].

In 2011, the FDA approved ipilimumab (Yervoy^™^), a human immunoglobulin (IgG1) antibody that binds and sequesters CTLA-4 with high affinity for treatment of unresectable or metastatic melanoma (Figure 4B). Ipilimumab significantly improved median overall survival for metastatic melanoma (10.1 vs. 6.4 months for control) in phase 3 clinical trials; on 5-year follow-up, 15–30% of ipilimumab treated patients were still alive, suggesting striking response durability [136]. Combination ipilimumab and fotemustine demonstrated 50% disease control for patients with brain metastases in a phase 2 trial, with a 12.7 month median survival and a 3-year survival rate of 27.8% [144]. In another study of patients with previously untreated metastatic melanoma, ipilimumab combined with dacarbazine extended survival beyond dacarbazine alone (median survival of 11.2 vs. 9.1 months, respectively) [142]. At 3 years, 20.8% of patients receiving combination ipilimumab/dacarbazine remained alive, compared to 12.2% for the dacarbazine arm. The almost 10% difference between survival curves for over 36 months corroborates the ability of ipilimumab to drive long-term response rates in a small proportion of patients.

A phase 2 multi-institutional trial evaluating the efficacy of high-dose ipilimumab (10 mg/kg 4x weekly) in intracranial melanoma demonstrated disease control in 24% of patients after three months, with 11% partial response, a portion of which were durable [145]. Of particular significance, these data suggest that ipilimumab in patients with CNS disease provides a similar measure of disease control as in those without CNS metastases; active intracranial disease had previously been excluded for all clinical trials with ipilimumab monotherapy. These findings indicate that ipilimumab may mitigate the poor prognosis of CNS disease, highlighting the systematic efficacy of immunotherapy.

Importantly, concomitant steroid use may also have an effect on patient response. Although steroids may be thought to improve certain symptoms, they also exert an immunosuppressive effect. In the referenced phase 2 ipilimumab trial [145], patients not requiring corticosteroids for clinical or radiological control displayed the most favorable outcomes (24% versus 10% response rate), suggesting that steroid use may negatively influence ipilimumab efficacy. Of note, however, the cohort requiring corticosteroids displayed more severe symptoms, which could partially account for the decrease in response rate.

Follow-up for 177 advanced melanoma patients enrolled in the earliest ipilimumab trials demonstrated median response duration of up to 7 years [143]. Notably, a proportion of initially reported partial responses progressed into complete responses after a prolonged period of time, with average complete response attained at 30 months after treatment initiation. This extensive temporal interval before achieving complete tumor response likely reflects the time necessary for T-cells to uncouple from CTLA-4 mediated inhibition, undergo activation, and subsequently infiltrate and eradicate the tumor. In support, melanoma biopsy after ipilimumab treatment shows clear evidence of tumor infiltrating lymphocytes (TIL) [146, 147].

Side effects of ipilimumab therapy include immune-mediated diarrhea, rash, adrenal insufficiency, and hypophysitis [148]. In a phase 3 trial of 676 patients, 14 deaths (2%) were drug-related, half of which were associated with immune-related adverse events [136]. The mechanistic role of CTLA-4 during early stages of T-cell activation—which leads to a more diffuse and nonspecific activation pattern compared to effector phase regulation—may explain the gamut of immune-triggered adverse events observed in clinical studies.

### PD-1 checkpoint inhibition

In comparison to the role of CTLA-4 early in T-cell activation, PD-1 and its ligands (PD-L1 and PD-L2) regulate the effector phase of T-cell activation (Figure 4A). PD-1 couples to ligand outside the lymph node in the peripheral tissues (e.g., the tumor cell microenvironment), leading to downregulation of effector function resulting in an “exhausted” phenotype and lymphocyte apoptosis [149]. PD-1 and PD-L1 expression colocalizes with TIL and circulating interferon-gamma in metastatic melanoma, suggesting a role in countering the host immune response [150]. Inhibition of the PD-1/PD-L1 axis *in vitro* reinstates T-cell eradication of melanoma cells [151]. PD-L1 expression is observed in approximately 47% of intracranial melanoma metastases [152].

Two PD-1 inhibitors have been approved by the FDA for the first-line treatment of metastatic melanoma (in September 2014 and January 2015, respectively): nivolumab (Opdivo^®^; Bristol-Myers Squibb, New York City, New York, USA) and pembrolizumab (formerly MK-3475 or lambrolizumab; Keytruda^®^; Merck, Kenilworth, New Jersey, USA). Nivolumab confers significant improvement to overall survival when compared with dacarbazine in previously untreated non-*BRAF*-mutant melanoma patients [153]. As with CTLA-4 inhibition, the duration of response to nivolumab appears to be relatively long-lived when compared to other therapeutic modalities. A phase 1 trial showed a mean overall response rate of 28% in patients with advanced, previously treated melanoma, with 62% experiencing a response durability of a year or longer [154]. During subsequent follow-up, the median OS was 16.8 months across all dosing regimens (0.1 mg/kg, 0.3 mg/kg, 1 mg/kg, 3 mg/kg, 10 mg/kg), with a peak of 20.3 months at the 3 mg/kg dose [155]. By targeting effector phase lymphocytes, PD-1 inhibition exhibits a more restricted pattern of immune upregulation compared with CTLA-4 suppression, which grants fewer immune-mediated side effects and a shorter time lag before complete tumor response than with ipilimumab, as TILs are already present in metastases.

Pembrolizumab also appears promising in metastatic melanoma, with measurable response rates observed in 37–38% of patients, tumor size reduction in 77%, and a median response durability of > 11 months at time of report [156]. In a 2016 trial, significant interim responses to anti-PD1 therapy in 39 patients with brain metastases were observed, with additional studies on intracranial melanomas presently underway [157].

Interestingly—and contrasting with molecular therapy—previous treatment with ipilimumab does not significantly affect the likelihood of mounting a sizeable tumor response with anti-PD1 therapy [156]. Additionally, severity of adverse events experienced with ipilimumab are not predictive of adverse events during second-line PD1 inhibition [158].

Studies evaluating PD-1 blocking antibodies specifically in patients with intracranial melanoma metastases are underway (NCT02374242, NCT02320058). Other anti-PD-1 antibodies in development include CT011 and PDR001 [159]. Expression of PD-1 ligand (PD-L1) by tumor lines is being investigated as a predictive biomarker with distinct genetic and morphological characteristics (e.g., increased aggressiveness) [160], with preliminary data suggesting that tumors lacking PD-L1 expression are less likely to respond to PD-1 inhibition compared to tumors expressing PD-L1 [161]. Several PD-L1 antibodies, including BMS-936559 and MPDL-3280A, are also under clinical testing. Initial evidence demonstrates that PD-L1 suppression induces durable melanoma regression, with an objective response rate of 17% at 6 months [162].

### Combinatorial immunotherapy

CTLA-4 and PD-1 co-inhibition offers an additional domain of clinical investigation. In an early clinical trial of combination nivolumab and ipilimumab in advanced melanoma patients, 53% of treatment-naïve patients had an objective response with rapid and extensive (80%) regression of tumor, although a number of adverse side effects—including hepatic, gastrointestinal, and renal events—were reported [163]. A more recent phase 1 study confirmed a response rate of 61% for ipilimumab/nivolumab combination therapy versus 11% for ipilimumab monotherapy, with complete response reported in 22% and 0%, respectively [164]. Accelerated approval of the combination for *BRAF*-mutant melanoma was granted by the FDA in January 2016. A phase 2 study investigating combination ipilimumab and nivolumab in active intracranial metastases is currently underway (NCT02320058).

Of note, the CTLA-4 and PD-1 immunological pathways are complementary and nonredundant, with CTLA-4 inhibition affecting early T-cell activation in lymph nodes and PD-1 suppression affecting effector T cells in peripheral tissue. Interestingly, in an animal model of melanoma, CTLA-4 inhibition resulted in a higher proportion of TIL expressing PD-1 while PD-1 inhibition led to increased TIL expression of CTLA-4 [165]. Treatment with CTLA-4 or PD-1 monotherapy may trigger upregulation of compensatory checkpoint mechanisms, highlighting the therapeutic potential of targeting multiple immunological pathways.

### Other immunotherapeutic treatments

Checkpoint inhibition is a recent evolution of long-standing experience with immunotherapy in melanoma patients. Interleukin-2 (IL-2) has been FDA-approved since 1998 for advanced melanoma therapy, based on data from phase 2 studies [166, 167]. High-dose administration is associated with overall response rates of 16%, with penetration to all sites of disease, including the CNS. Median response durability is greater than six months; in 5% of patients with metastatic spread, clinical benefit may be observed for decades [168]. At high concentrations, however, IL-2 is significantly toxic, causing capillary leak syndrome and increased cerebral edema, thereby restricting its therapeutic profile to patients without cardiopulmonary morbidities and with good performance status.

Adoptive cell transfer (ACT), another immunotherapy strategy, involves collection of lymphocytes, either TILs or blood T cells, from the patient, followed by *ex vivo* activation and expansion (e.g., through the use of chimeric antigen receptors (CAR), which are used to graft disease-targeted monoclonal antibodies onto T cells), and subsequent infusion of processed cells back into the patient to induce an immune response [169]. In a recent report of ACT in metastatic melanoma patients, an overall response rate of 56% was observed in 93 patients [170]. No relapses were reported within a follow-up of 31 months among the 11% who achieved a complete response.

**Figure 5:**
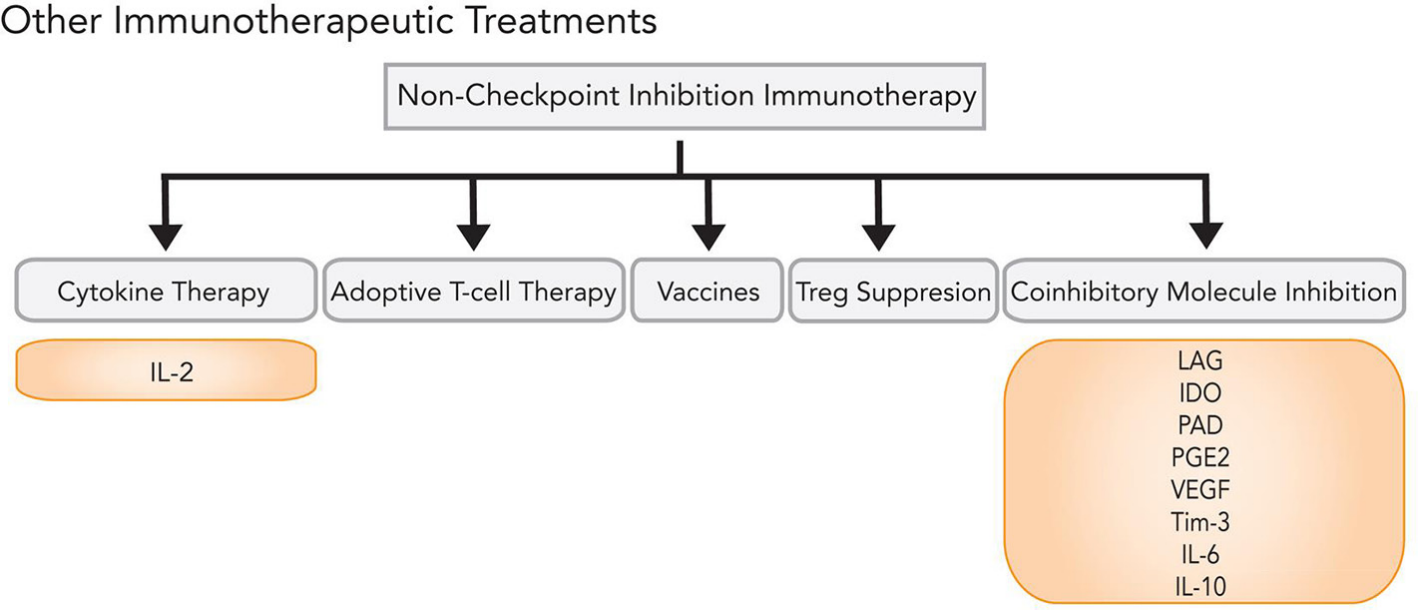
Immunotherapy options beyond checkpoint inhibition

In addition, inhibition of auxiliary coinhibitory molecules—such as lymphocyte activation gene 3 (LAG-3), indoleamine-2,3-dioxygenase (IDO), arginine deaminase, prostaglandin E2, VEGF, T-cell immunoglobulin domain and mucin domain 3 (TIM-3), IL-6, IL-10, and other cytokines—are currently in clinical trials (Figure 5). Preliminary clinical results have also suggested the plausibility of novel immunotherapeutic combinations, including ipilimumab and high-dose IL-2 [143]. Other potential complementary therapies include vaccines and regulatory T-cell suppression. For intracranial melanoma metastases, combination of immunotherapeutic treatment with adjuvant SRS synergistically improves BBB permeability, stimulates cytokine release, and increases antigen presentation on melanoma surface cells, with durability rates of > 4 years [171–173].

### Resistance mechanisms of immune escape

Resistance via immune escape has been reported for various immunotherapeutic treatments, although less frequently than with BRAF inhibition. Potential mechanisms include selection of antigen-deficient tumor variants, induction of T-cell tolerance, antigen loss, and inflammation-induced reversible loss of melanocytic antigens [63, 174]. Melanoma-associated antigens include tumor-associated testis-specific antigens (e.g., MAGE, BAGE, and GAGE), melanocyte differentiation antigens (e.g., Melan-A/MART-1, tyrosinase), and aberrantly expressed or mutated molecules (e.g., CDK4, beta-catenin) [175].

In conditions of increased lymphocytic cytotoxicity against melanoma antigens, a portion of tumor cells switch from a differentiated to a dedifferentiated phenotype in response to T-cell driven inflammation [176, 177]. This process is mediated by tumor necrosis factor alpha (TNFα), which camouflages tumor cells against the immune system and is reversible upon discontinuation of therapy and resolution of inflammation. Upregulation of immunosuppressive mechanisms in melanoma may also inhibit T-cell activation, including IDO, PD-L1/B7-H1, and FoxP3+ regulatory T cells [178]. These factors serve as negative feedback mechanisms following T-cell infiltration, suggesting suppressive regulatory immune checkpoints as viable future clinical targets.

*In vitro* experiments have also demonstrated a role for apoptosis regulation as a means of modulating tumor resistance, through downregulation of death receptors (DRs) and the TNF-related apoptosis-inducing ligand (TRAIL) cytotoxic pathway [179]. Resistant melanoma cell lines demonstrate DR5 downregulation and an inverted proportion of pro- versus anti-apoptotic molecules, the effects of which can be countered by histone deacetylase (HDAC) inhibitors. This category of compounds prevents silencing of pro-apoptotic genes by blocking HDAC from deacetylating histone moieties. One such drug, vorinostat, has received FDA approval for the treatment of cutaneous T-cell lymphoma. Delivery of chromatin remodeling drugs to immune-resistant melanomas may be able to counter acquired resistance by shifting the intracellular climate towards a pro-apoptotic status through increased DR expression. Indomethacin, a non-steroidal anti-inflammatory drug, has also been shown to enhance TRAIL-mediated apoptosis through upregulation of DR5 and down-modulation of survivin in human melanoma cell lines [180].

Additional modalities of acquired resistance include mechanisms that interfere with T-lymphocyte activity within the melanoma microenvironment [181]. Multiple checkpoint molecules have been observed to dampen the immune response, including LAG-3, TIM-3, and B- and T-lymphocyte attenuator (BTLA) [182]. Immunotherapy may also confer a fitness advantage to melanoma subpopulations with loss of MHC expression, which renders the tumor effectually invisible to the adaptive immune system [63, 183].

Unlike with BRAF and MEK inhibitors, treatment failure with one immunotherapy does not preclude response to others; patients resistant to ipilimumab can still have a response to anti-PD-1, and vice versa [156, 184]. Interestingly, retreatment is possible in tumor recurrences [185, 186], suggesting the activation and expansion of a new cohort of T-cells specific to the evolving tumor antigen repertoire.

Current knowledge regarding clinical benefits of immunotherapy may provide guidance for future trials of novel agents with respect to metastatic melanoma. Drugs with low response rates sustained by intratumoral lymphocyte infiltration—e.g., anti-CTLA4 antibodies—should focus on response durability as the main trial endpoint [187]; agents with high response rates and well-characterized mechanisms should focus on clinically meaningful duration. Combination immunotherapies, in turn, should focus on increased durability of response rates after addition of the co-therapeutic agent. Pharmacokinetic aspects relative to tumor penetrance, drug concentration in tumor cells, molecular stability, and receptor occupancy should also be noted. Improved characterization of drugs under development may accelerate and streamline trial design and meaningful interpretation of clinical endpoints.

## CONCLUSIONS

Since 2011, seven novel agents have been introduced for targeted treatment of late-stage melanoma, including patients with intracranial disease—vemurafenib, dabrafenib, trametinib, cobimetinib, ipilimumab, nivolumab, and pembrolizumab. Therapeutic choices should be personalized based on multiple factors: *BRAF* mutational status, comorbidities, tumor burden, and immune status.

Current consensus in the literature indicates that patients with *BRAF* mutant brain tumors should receive a *BRAF* inhibitor or combinatorial therapy with BRAF and MEK co-inhibition. There is no definitive clinical evidence suggesting that one inhibitor outperforms the other, although consideration should be taken regarding side effects. The rising epidemic of *BRAF* inhibitor resistance, as a consequence, invokes increasing combinatorial strategies for treatment of *BRAF*-mutant metastatic melanoma. Immunotherapy offers additional treatment options in exploiting the endogenous immune system to destroy metastatic tumor, with strikingly durable responses of several years in some. In addition, conventional treatments such as surgery and radiation continue to play a role in control of tumor progression for select clinical indications.

Despite the new ammunition against metastatic melanoma over the past five years, prognosis remains guarded. Further investigations into countering resistance, minimizing toxicity, optimizing combination therapies, and improving survival remain critically needed. By formulating a better framework for understanding the efficacy and limitations of current therapeutic modalities, improved prognoses and disease management may be attainable for intracranial melanoma diagnoses.

## References

[1] Miller AJ, Mihm MC (2006). Melanoma. N Engl J Med.

[2] Fiddler IJ (1995). Melanoma Metastasis. Cancer Control.

[3] Balch CM, Gershenwald JE, Soong SJ, Thompson JF, Atkins MB, Byrd DR, Buzaid AC, Cochran AJ, Coit DG, Ding S, Eggermont AM, Flaherty KT, Gimotty PA (2009). Final version of 2009 AJCC melanoma staging and classification. J Clin Oncol.

[4] Kim C, Lee CW, Kovacic L, Shah A, Klasa R, Savage KJ (2010). Long-term survival in patients with metastatic melanoma treated with DTIC or temozolomide. Oncologist.

[5] Staudt M, Lasithiotakis K, Leiter U, Meier F, Eigentler T, Bamberg M, Tatagiba M, Brossart P, Garbe C (2010). Determinants of survival in patients with brain metastases from cutaneous melanoma. Br J Cancer.

[6] Davies MA, Liu P, McIntyre S, Kim KB, Papadopoulos N, Hwu WJ, Hwu P, Bedikian A (2011). Prognostic factors for survival in melanoma patients with brain metastases. Cancer.

[7] Sampson JH, Carter JH, Friedman AH, Seigler HF (1998). Demographics, prognosis, and therapy in 702 patients with brain metastases from malignant melanoma. J Neurosurg.

[8] Papadatos-Pastos D, Soultati A, Harries M (2013). Targeting brain metastases in patients with melanoma. Biomed Res Int.

[9] Hanson PW, Elaimy AL, Lamoreaux WT, Demakas JJ, Fairbanks RK, Mackay AR, Taylor B, Cooke BS, Thumma SR, Lee CM (2012). A concise review of the efficacy of stereotactic radiosurgery in the management of melanoma and renal cell carcinoma brain metastases. World J Surg Oncol.

[10] Markovic SN, Erickson LA, Rao RD, Weenig RH, Pockaj BA, Bardia A, Vachon CM, Schild SE, McWilliams RR, Hand JL, Laman SD, Kottschade LA, Maples WJ (2007). Malignant melanoma in the 21st century, part 1: epidemiology, risk factors, screening, prevention, and diagnosis. Mayo Clin Proc.

[11] Linos E, Swetter SM, Cockburn MG, Colditz GA, Clarke CA (2009). Increasing burden of melanoma in the United States. J Invest Dermatol.

[12] Curtin JA, Fridlyand J, Kageshita T, Patel HN, Busam KJ, Kutzner H, Cho KH, Aiba S, Brocker EB, LeBoit PE, Pinkel D, Bastian BC (2005). Distinct sets of genetic alterations in melanoma. N Engl J Med.

[13] Posner JB, Chernik NL (1978). Intracranial metastases from systemic cancer. Adv Neurol.

[14] Zimm S, Wampler GL, Stablein D, Hazra T, Young HF (1981). Intracerebral metastases in solid-tumor patients: natural history and results of treatment. Cancer.

[15] Johnson JD, Young B (1996). Demographics of brain metastasis. Neurosurg Clin N Am.

[16] Suranagi VV, Maste P, Malur PR (2015). Primary intracranial malignant melanoma: a rare case with review of literature. Asian J Neurosurg.

[17] Spanknebel K, Kaufman HL (2004). Surgical treatment of stage IV melanoma. Clin Dermatol.

[18] Gorantla V, Kirkwood JM, Tawbi HA (2013). Melanoma brain metastases: an unmet challenge in the era of active therapy. Curr Oncol Rep.

[19] Gummadi T, Zhang BY, Valpione S, Kim C, Kottschade LA, Mittapalli RK, Chiarion-Sileni V, Pigozzo J, Elmquist WF, Dudek AZ (2015). Impact of BRAF mutation and BRAF inhibition on melanoma brain metastases. Melanoma Res.

[20] Zakrzewski J, Geraghty LN, Rose AE, Christos PJ, Mazumdar M, Polsky D, Shapiro R, Berman R, Darvishian F, Hernando E, Pavlick A, Osman I (2011). Clinical variables and primary tumor characteristics predictive of the development of melanoma brain metastases and post-brain metastases survival. Cancer.

[21] Litvak DA, Gupta RK, Yee R, Wanek LA, Ye W, Morton DL (2004). Endogenous immune response to early- and intermediate-stage melanoma is correlated with outcomes and is independent of locoregional relapse and standard prognostic factors. J Am Coll Surg.

[22] Kammerer PW, Shabazfar N, Palarie V, Kleis W, Al-Nawas B (2011). Therapy and prognosis of extraoral malignant melanoma metastasizing to the jaw: case report and literature review. J Oral Maxillofac Surg.

[23] Andrews DW, Scott CB, Sperduto PW, Flanders AE, Gaspar LE, Schell MC, Werner-Wasik M, Demas W, Ryu J, Bahary JP, Souhami L, Rotman M, Mehta MP (2004). Whole brain radiation therapy with or without stereotactic radiosurgery boost for patients with one to three brain metastases: phase III results of the RTOG 9508 randomised trial. Lancet.

[24] Kalkanis SN, Kondziolka D, Gaspar LE, Burri SH, Asher AL, Cobbs CS, Ammirati M, Robinson PD, Andrews DW, Loeffler JS, McDermott M, Mehta MP, Mikkelsen T (2010). The role of surgical resection in the management of newly diagnosed brain metastases: a systematic review and evidence-based clinical practice guideline. J Neurooncol.

[25] Kocher M, Soffietti R, Abacioglu U, Villa S, Fauchon F, Baumert BG, Fariselli L, Tzuk-Shina T, Kortmann RD, Carrie C, Ben Hassel M, Kouri M, Valeinis E (2011). Adjuvant whole-brain radiotherapy versus observation after radiosurgery or surgical resection of one to three cerebral metastases: results of the EORTC 22952–26001 study. J Clin Oncol.

[26] Patchell RA, Tibbs PA, Regine WF, Dempsey RJ, Mohiuddin M, Kryscio RJ, Markesbery WR, Foon KA, Young B (1998). Postoperative radiotherapy in the treatment of single metastases to the brain: a randomized trial. JAMA.

[27] Mori Y, Kondziolka D, Flickinger JC, Kirkwood JM, Agarwala S, Lunsford LD (1998). Stereotactic radiosurgery for cerebral metastatic melanoma: factors affecting local disease control and survival. Int J Radiat Oncol Biol Phys.

[28] Mathieu D, Kondziolka D, Cooper PB, Flickinger JC, Niranjan A, Agarwala S, Kirkwood J, Lunsford LD (2007). Gamma knife radiosurgery for malignant melanoma brain metastases. Clin Neurosurg.

[29] Liew DN, Kano H, Kondziolka D, Mathieu D, Niranjan A, Flickinger JC, Kirkwood JM, Tarhini A, Moschos S, Lunsford LD (2011). Outcome predictors of gamma knife surgery for melanoma brain metastases. J Neurosurg.

[30] Yamamoto M, Serizawa T, Shuto T, Akabane A, Higuchi Y, Kawagishi J, Yamanaka K, Sato Y, Jokura H, Yomo S, Nagano O, Kenai H, Moriki A (2014). Stereotactic radiosurgery for patients with multiple brain metastases (JLGK0901): a multi-institutional prospective observational study. Lancet Oncol.

[31] Dyer MA, Arvold ND, Chen YH, Pinnell NE, Mitin T, Lee EQ, Hodi FS, Ibrahim N, Weiss SE, Kelly PJ, Floyd SR, Mahadevan A, Alexander BM (2014). The role of whole brain radiation therapy in the management of melanoma brain metastases. Radiat Oncol.

[32] Rades D, Panzner A, Dziggel L, Haatanen T, Lohynska R, Schild SE (2012). Dose-escalation of whole-brain radiotherapy for brain metastasis in patients with a favorable survival prognosis. Cancer.

[33] Auchter RM, Lamond JP, Alexander E, Buatti JM, Chappell R, Friedman WA, Kinsella TJ, Levin AB, Noyes WR, Schultz CJ, Loeffler JS, Mehta MP (1996). A multiinstitutional outcome and prognostic factor analysis of radiosurgery for resectable single brain metastasis. Int J Radiat Oncol Biol Phys.

[34] Li J, Bentzen SM, Li J, Renschler M, Mehta MP (2008). Relationship between neurocognitive function and quality of life after whole-brain radiotherapy in patients with brain metastasis. Int J Radiat Oncol Biol Phys.

[35] Sperduto PW, Chao ST, Sneed PK, Luo X, Suh J, Roberge D, Bhatt A, Jensen AW, Brown PD, Shih H, Kirkpatrick J, Schwer A, Gaspar LE (2010). Diagnosis-specific prognostic factors, indexes, and treatment outcomes for patients with newly diagnosed brain metastases: a multi-institutional analysis of 4,259 patients. Int J Radiat Oncol Biol Phys.

[36] Broadbent AM, Hruby G, Tin MM, Jackson M, Firth I (2004). Survival following whole brain radiation treatment for cerebral metastases: an audit of 474 patients. Radiother Oncol.

[37] Meier S, Baumert BG, Maier T, Wellis G, Burg G, Seifert B, Dummer R (2004). Survival and prognostic factors in patients with brain metastases from malignant melanoma. Onkologie.

[38] Fife KM, Colman MH, Stevens GN, Firth IC, Moon D, Shannon KF, Harman R, Petersen-Schaefer K, Zacest AC, Besser M, Milton GW, McCarthy WH, Thompson JF (2004). Determinants of outcome in melanoma patients with cerebral metastases. J Clin Oncol.

[39] Comis RL (1976). DTIC (NSC-45388) in malignant melanoma: a perspective. Cancer Treat Rep.

[40] Atkins MB, Kunkel L, Sznol M, Rosenberg SA (2000). High-dose recombinant interleukin-2 therapy in patients with metastatic melanoma: long-term survival update. Cancer J Sci Am.

[41] Serrone L, Zeuli M, Sega FM, Cognetti F (2000). Dacarbazine-based chemotherapy for metastatic melanoma: thirty-year experience overview. J Exp Clin Cancer Res.

[42] Middleton MR, Grob JJ, Aaronson N, Fierlbeck G, Tilgen W, Seiter S, Gore M, Aamdal S, Cebon J, Coates A, Dreno B, Henz M, Schadendorf D (2000). Randomized phase III study of temozolomide versus dacarbazine in the treatment of patients with advanced metastatic malignant melanoma. J Clin Oncol.

[43] Bedikian AY, Millward M, Pehamberger H, Conry R, Gore M, Trefzer U, Pavlick AC, DeConti R, Hersh EM, Hersey P, Kirkwood JM, Haluska FG, Oblimersen Melanoma Study G (2006). Bcl-2 antisense (oblimersen sodium) plus dacarbazine in patients with advanced melanoma: the Oblimersen Melanoma Study Group. J Clin Oncol.

[44] de Vries NA, Zhao J, Kroon E, Buckle T, Beijnen JH, van Tellingen O (2007). P-glycoprotein and breast cancer resistance protein: two dominant transporters working together in limiting the brain penetration of topotecan. Clin Cancer Res.

[45] Addeo R, Zappavigna S, Luce A, Facchini S, Caraglia M (2013). Chemotherapy in the management of brain metastases: the emerging role of fotemustine for patients with melanoma and NSCLC. Expert Opin Drug Saf.

[46] Margolin K, Atkins B, Thompson A, Ernstoff S, Weber J, Flaherty L, Clark I, Weiss G, Sosman J, Smith W, Dutcher P, Gollob J, Longmate J (2002). Temozolomide and whole brain irradiation in melanoma metastatic to the brain: a phase II trial of the Cytokine Working Group. J Cancer Res Clin Oncol.

[47] Avril MF, Aamdal S, Grob JJ, Hauschild A, Mohr P, Bonerandi JJ, Weichenthal M, Neuber K, Bieber T, Gilde K, Guillem Porta V, Fra J, Bonneterre J (2004). Fotemustine compared with dacarbazine in patients with disseminated malignant melanoma: a phase III study. J Clin Oncol.

[48] Zhu W, Zhou L, Qian JQ, Qiu TZ, Shu YQ, Liu P (2014). Temozolomide for treatment of brain metastases: A review of 21 clinical trials. World J Clin Oncol.

[49] Tawbi HA, Beumer JH, Tarhini AA, Moschos S, Buch SC, Egorin MJ, Lin Y, Christner S, Kirkwood JM (2013). Safety and efficacy of decitabine in combination with temozolomide in metastatic melanoma: a phase I/II study and pharmacokinetic analysis. Ann Onc.

[50] Trinh VA, Hwu WJ (2012). Chemoprevention for brain metastases. Curr Onc Rep.

[51] Preusser M, Berghoff AS, Schadendorf D, Lin NU, Stupp R (2012). Brain metastasis: opportunity for drug development?. Curr Opin Neurol.

[52] Brewster AM, Davidson NE, McCaskill-Stevens W (2012). Chemoprevention for breast cancer: overcoming barriers to treatment. Am Soc Clin Oncol Educ Book.

[53] Swanton C (2012). Intratumor heterogeneity: evolution through space and time. Cancer Res.

[54] Shields JD, Borsetti M, Rigby H, Harper SJ, Mortimer PS, Levick JR, Orlando A, Bates DO (2004). Lymphatic density and metastatic spread in human malignant melanoma. Br J Cancer.

[55] Payne AS, Cornelius LA (2002). The role of chemokines in melanoma tumor growth and metastasis. J Invest Dermatol.

[56] Denkins Y, Reiland J, Roy M, Sinnappah-Kang ND, Galjour J, Murry BP, Blust J, Aucoin R, Marchetti D (2004). Brain metastases in melanoma: roles of neurotrophins. Neuro Oncol.

[57] Truzzi F, Marconi A, Lotti R, Dallaglio K, French LE, Hempstead BL, Pincelli C (2008). Neurotrophins and their receptors stimulate melanoma cell proliferation and migration. J Invest Dermatol.

[58] Murakami T, Cardones AR, Hwang ST (2004). Chemokine receptors and melanoma metastasis. J Dermatol Sci.

[59] Jilaveanu LB, Parisi F, Barr ML, Zito CR, Cruz-Munoz W, Kerbel RS, Rimm DL, Bosenberg MW, Halaban R, Kluger Y, Kluger HM (2015). PLEKHA5 as a biomarker and potential mediator of melanoma brain metastasis. Clin Cancer Res.

[60] Kusters B, Leenders WP, Wesseling P, Smits D, Verrijp K, Ruiter DJ, Peters JP, van Der Kogel AJ, de Waal RM (2002). Vascular endothelial growth factor-A(165) induces progression of melanoma brain metastases without induction of sprouting angiogenesis. Cancer Res.

[61] Huang FJ, Steeg PS, Price JE, Chiu WT, Chou PC, Xie K, Sawaya R, Huang S (2008). Molecular basis for the critical role of suppressor of cytokine signaling-1 in melanoma brain metastasis. Cancer Res.

[62] Zhang L, Zhang S, Yao J, Lowery FJ, Zhang Q, Huang WC, Li P, Li M, Wang X, Zhang C, Wang H, Ellis K, Cheerathodi M (2015). Microenvironment-induced PTEN loss by exosomal microRNA primes brain metastasis outgrowth. Nature.

[63] Dunn GP, Bruce AT, Ikeda H, Old LJ, Schreiber RD (2002). Cancer immunoediting: from immunosurveillance to tumor escape. Nature Immunol.

[64] Dunn GP, Okada H (2015). Principles of immunology and its nuances in the central nervous system. Neuro Oncol.

[65] Cserr HF, Harling-Berg CJ, Knopf PM (1992). Drainage of brain extracellular fluid into blood and deep cervical lymph and its immunological significance. Brain Pathol.

[66] Clemente CG, Mihm MC, Bufalino R, Zurrida S, Collini P, Cascinelli N (1996). Prognostic value of tumor infiltrating lymphocytes in the vertical growth phase of primary cutaneous melanoma. Cancer.

[67] Mihm MC, Clemente CG, Cascinelli N (1996). Tumor infiltrating lymphocytes in lymph node melanoma metastases: a histopathologic prognostic indicator and an expression of local immune response. Lab Invest.

[68] Louveau A, Smirnov I, Keyes TJ, Eccles JD, Rouhani SJ, Peske JD, Derecki NC, Castle D, Mandell JW, Lee KS, Harris TH, Kipnis J (2015). Structural and functional features of central nervous system lymphatic vessels. Nature.

[69] Aspelund A, Antila S, Proulx ST, Karlsen TV, Karaman S, Detmar M, Wiig H, Alitalo K (2015). A dural lymphatic vascular system that drains brain interstitial fluid and macromolecules. J Exp Med.

[70] Flaherty KT, Puzanov I, Kim KB, Ribas A, McArthur GA, Sosman JA, O'Dwyer PJ, Lee RJ, Grippo JF, Nolop K, Chapman PB (2010). Inhibition of mutated, activated BRAF in metastatic melanoma. N Engl J Med.

[71] Chapman PB, Hauschild A, Robert C, Haanen JB, Ascierto P, Larkin J, Dummer R, Garbe C, Testori A, Maio M, Hogg D, Lorigan P, Lebbe C (2011). Improved survival with vemurafenib in melanoma with BRAF V600E mutation. N Engl J Med.

[72] Wolchok JD, Neyns B, Linette G, Negrier S, Lutzky J, Thomas L, Waterfield W, Schadendorf D, Smylie M, Guthrie T, Grob JJ, Chesney J (2010). Ipilimumab monotherapy in patients with pretreated advanced melanoma: a randomised, double-blind, multicentre, phase 2, dose-ranging study. Lancet Oncol.

[73] Lyle M, Long GV (2014). The role of systemic therapies in the management of melanoma brain metastases. Curr Opin Oncol.

[74] Davies H, Bignell GR, Cox C, Stephens P, Edkins S, Clegg S, Teague J, Woffendin H, Garnett MJ, Bottomley W, Davis N, Dicks E, Ewing R (2002). Mutations of the BRAF gene in human cancer. Nature.

[75] Jakob JA, Bassett RL, Ng CS, Curry JL, Joseph RW, Alvarado GC, Rohlfs ML, Richard J, Gershenwald JE, Kim KB, Lazar AJ, Hwu P (2012). NRAS mutation status is an independent prognostic factor in metastatic melanoma. Cancer.

[76] Greaves WO, Verma S, Patel KP, Davies MA, Barkoh BA, Galbincea JM, Yao H, Lazar AJ, Aldape KD, Medeiros LJ, Luthra R (2013). Frequency and spectrum of BRAF mutations in a retrospective, single-institution study of 1112 cases of melanoma. J Mol Diagn.

[77] Bucheit AD, Syklawer E, Jakob JA, Bassett RL, Curry JL, Gershenwald JE, Kim KB, Hwu P, Lazar AJ, Davies MA (2013). Clinical characteristics and outcomes with specific BRAF and NRAS mutations in patients with metastatic melanoma. Cancer.

[78] Menzies AM, Haydu LE, Visintin L, Carlino MS, Howle JR, Thompson JF, Kefford RF, Scolyer RA, Long GV (2012). Distinguishing clinicopathologic features of patients with V600E and V600K BRAF-mutant metastatic melanoma. Clin Cancer Res.

[79] Gorden A, Osman I, Gai W, He D, Huang W, Davidson A, Houghton AN, Busam K, Polsky D (2003). Analysis of BRAF and N-RAS mutations in metastatic melanoma tissues. Cancer Res.

[80] Viros A, Sanchez-Laorden B, Pedersen M, Furney SJ, Rae J, Hogan K, Ejiama S, Girotti MR, Cook M, Dhomen N, Marais R (2014). Ultraviolet radiation accelerates BRAF-driven melanomagenesis by targeting TP53. Nature.

[81] Brady DC, Crowe MS, Turski ML, Hobbs GA, Yao X, Chaikuad A, Knapp S, Xiao K, Campbell SL, Thiele DJ, Counter CM (2014). Copper is required for oncogenic BRAF signalling and tumorigenesis. Nature.

[82] Macerola E, Loggini B, Giannini R, Garavello G, Giordano M, Proietti A, Niccoli C, Basolo F, Fontanini G (2015). Coexistence of TERT promoter and BRAF mutations in cutaneous melanoma is associated with more clinicopathological features of aggressiveness. Virchows Arch.

[83] Sosman JA, Kim KB, Schuchter L, Gonzalez R, Pavlick AC, Weber JS, McArthur GA, Hutson TE, Moschos SJ, Flaherty KT, Hersey P, Kefford R, Lawrence D (2012). Survival in BRAF V600-mutant advanced melanoma treated with vemurafenib. N Engl J Med.

[84] Young K, Minchom A, Larkin J (2012). BRIM-1, -2 and -3 trials: improved survival with vemurafenib in metastatic melanoma patients with a BRAF(V600E) mutation. Future Oncol.

[85] Rochet NM, Kottschade LA, Markovic SN (2011). Vemurafenib for melanoma metastases to the brain. N Engl J Med.

[86] Dummer R, Goldinger SM, Turtschi CP, Eggmann NB, Michielin O, Mitchell L, Veronese L, Hilfiker PR, Felderer L, Rinderknecht JD (2014). Vemurafenib in patients with BRAF(V600) mutation-positive melanoma with symptomatic brain metastases: final results of an open-label pilot study. Eur J Cancer.

[87] Peuvrel L, Saint-Jean M, Quereux G, Brocard A, Khammari A, Knol AC, Dreno B (2014). Incidence and characteristics of melanoma brain metastases developing during treatment with vemurafenib. J Neurooncol.

[88] Long GV, Trefzer U, Davies MA, Kefford RF, Ascierto PA, Chapman PB, Puzanov I, Hauschild A, Robert C, Algazi A, Mortier L, Tawbi H, Wilhelm T (2012). Dabrafenib in patients with Val600Glu or Val600Lys BRAF-mutant melanoma metastatic to the brain (BREAK-MB): a multicentre, open-label, phase 2 trial. Lancet Oncol.

[89] Hauschild A, Grob JJ, Demidov LV, Jouary T, Gutzmer R, Millward M, Rutkowski P, Blank CU, Miller WH, Kaempgen E, Martin-Algarra S, Karaszewska B (2012). Dabrafenib in BRAF-mutated metastatic melanoma: a multicentre, open-label, phase 3 randomised controlled trial. Lancet.

[90] Agarwala SS, Kirkwood JM, Gore M, Dreno B, Thatcher N, Czarnetski B, Atkins M, Buzaid A, Skarlos D, Rankin EM (2004). Temozolomide for the treatment of brain metastases associated with metastatic melanoma: a phase II study. J Clin Oncol.

[91] Long GV, Margolin KA (2013). Multidisciplinary approach to brain metastasis from melanoma: the emerging role of systemic therapies. Am Soc Clin Oncol Educ Book.

[92] Mittapalli RK, Vaidhyanathan S, Dudek AZ, Elmquist WF (2013). Mechanisms limiting distribution of the threonine-protein kinase B-RaF(V600E) inhibitor dabrafenib to the brain: implications for the treatment of melanoma brain metastases. J Pharmacol Exp Ther.

[93] Mittapalli RK, Vaidhyanathan S, Sane R, Elmquist WF (2012). Impact of P-glycoprotein (ABCB1) and breast cancer resistance protein (ABCG2) on the brain distribution of a novel BRAF inhibitor: vemurafenib (PLX4032). J Pharmacol Exp Ther.

[94] Mavropoulos JC, Wang TS (2014). Managing the skin toxicities from new melanoma drugs. Curr Treat Options Oncol.

[95] Gibney GT, Messina JL, Fedorenko IV, Sondak VK, Smalley KS (2013). Paradoxical oncogenesis—the long-term effects of BRAF inhibition in melanoma. Nat Rev Clin Oncol.

[96] Manousaridis I, Mavridou S, Goerdt S, Leverkus M, Utikal J (2013). Cutaneous side effects of inhibitors of the RAS/RAF/MEK/ERK signalling pathway and their management. J Eur Acad Dermatol Venereol.

[97] Oberholzer PA, Kee D, Dziunycz P, Sucker A, Kamsukom N, Jones R, Roden C, Chalk CJ, Ardlie K, Palescandolo E, Piris A, MacConaill LE, Robert C (2012). RAS mutations are associated with the development of cutaneous squamous cell tumors in patients treated with RAF inhibitors. J Clin Oncol.

[98] Solit DB, Garraway LA, Pratilas CA, Sawai A, Getz G, Basso A, Ye Q, Lobo JM, She Y, Osman I, Golub TR, Sebolt-Leopold J, Sellers WR (2006). BRAF mutation predicts sensitivity to MEK inhibition. Nature.

[99] Ascierto PA, Schadendorf D, Berking C, Agarwala SS, van Herpen CM, Queirolo P, Blank CU, Hauschild A, Beck JT, St-Pierre A, Niazi F, Wandel S, Peters M (2013). MEK162 for patients with advanced melanoma harbouring NRAS or Val600 BRAF mutations: a non-randomised, open-label phase 2 study. Lancet Oncol.

[100] Flaherty KT, Robert C, Hersey P, Nathan P, Garbe C, Milhem M, Demidov LV, Hassel JC, Rutkowski P, Mohr P, Dummer R, Trefzer U, Larkin JM (2012). Improved survival with MEK inhibition in BRAF-mutated melanoma. N Engl J Med.

[101] Long GV, Stroyakovsky DL, Gogas H, Levchenko E, De Braud F, Larkin J, Garbe C, Jouary T, Hauschild A, Grob JJ, Chiarion Sileni V, Lebbe C, Mandala M (2015). COMBI-d: A randomized, double-blinded, Phase III study comparing the combination of dabrafenib and trametinib to dabrafenib and trametinib placebo as first-line therapy in patients (pts) with unresectable or metastatic BRAFV600E/Kmutation-positive cutaneous melanoma. J Clin Oncol.

[102] Larkin J, Ascierto PA, Dreno B, Atkinson V, Liszkay G, Maio M, Mandala M, Demidov L, Stroyakovskiy D, Thomas L, de la Cruz-Merino L, Dutriaux C, Garbe C (2014). Combined vemurafenib and cobimetinib in BRAF-mutated melanoma. N Engl J Med.

[103] Vaidhyanathan S, Mittapalli RK, Sarkaria JN, Elmquist WF (2014). Factors influencing the CNS distribution of a novel MEK-1/2 inhibitor: implications for combination therapy for melanoma brain metastases. Drug Metab Dispos.

[104] Giroux S (2013). Overcoming acquired resistance to kinase inhibition: the cases of EGFR, ALK and BRAF. Bioorg Med Chem Lett.

[105] Wagle N, Emery C, Berger MF, Davis MJ, Sawyer A, Pochanard P, Kehoe SM, Johannessen CM, Macconaill LE, Hahn WC, Meyerson M, Garraway LA (2011). Dissecting therapeutic resistance to RAF inhibition in melanoma by tumor genomic profiling. J Clin Oncol.

[106] Nazarian R, Shi H, Wang Q, Kong X, Koya RC, Lee H, Chen Z, Lee MK, Attar N, Sazegar H, Chodon T, Nelson SF, McArthur G (2010). Melanomas acquire resistance to B-RAF(V600E) inhibition by RTK or N-RAS upregulation. Nature.

[107] Shi H, Hugo W, Kong X, Hong A, Koya RC, Moriceau G, Chodon T, Guo R, Johnson DB, Dahlman KB, Kelley MC, Kefford RF, Chmielowski B (2014). Acquired resistance and clonal evolution in melanoma during BRAF inhibitor therapy. Cancer Discov.

[108] Poulikakos PI, Persaud Y, Janakiraman M, Kong X, Ng C, Moriceau G, Shi H, Atefi M, Titz B, Gabay MT, Salton M, Dahlman KB, Tadi M (2011). RAF inhibitor resistance is mediated by dimerization of aberrantly spliced BRAF(V600E). Nature.

[109] Van Allen EM, Wagle N, Sucker A, Treacy DJ, Johannessen CM, Goetz EM, Place CS, Taylor-Weiner A, Whittaker S, Kryukov GV, Hodis E, Rosenberg M, McKenna A (2014). The genetic landscape of clinical resistance to RAF inhibition in metastatic melanoma. Cancer Discov.

[110] Montagut C, Sharma SV, Shioda T, McDermott U, Ulman M, Ulkus LE, Dias-Santagata D, Stubbs H, Lee DY, Singh A, Drew L, Haber DA, Settleman J (2008). Elevated CRAF as a potential mechanism of acquired resistance to BRAF inhibition in melanoma. Cancer Res.

[111] Boussemart L, Malka-Mahieu H, Girault I, Allard D, Hemmingsson O, Tomasic G, Thomas M, Basmadjian C, Ribeiro N, Thuaud F, Mateus C, Routier E, Kamsu-Kom N (2014). eIF4F is a nexus of resistance to anti-BRAF and anti-MEK cancer therapies. Nature.

[112] Sun C, Wang L, Huang S, Heynen GJ, Prahallad A, Robert C, Haanen J, Blank C, Wesseling J, Willems SM, Zecchin D, Hobor S, Bajpe PK (2014). Reversible and adaptive resistance to BRAF(V600E) inhibition in melanoma. Nature.

[113] Daud A, Weber JS, Sosman JA (2014). Overall Survival (OS) Update for BRF113220 Part C, a Phase II three-arm randomized study of dabrafenib (D) alone vs. combination of dabrafenib and trametinib (D+T) in pts with BRAF V600 mutation-positive (+) metastatic melanoma (MM). J Clin Oncol.

[114] Long GV, Stroyakovskiy D, Gogas H, Levchenko E, de Braud F, Larkin J, Garbe C, Jouary T, Hauschild A, Grob JJ, Chiarion Sileni V, Lebbe C, Mandala M (2014). Combined BRAF and MEK inhibition versus BRAF inhibition alone in melanoma. N Engl J Med.

[115] Kim KB, Kefford R, Pavlick AC, Infante JR, Ribas A, Sosman JA, Fecher LA, Millward M, McArthur GA, Hwu P, Gonzalez R, Ott PA, Long GV (2013). Phase II study of the MEK1/MEK2 inhibitor Trametinib in patients with metastatic BRAF-mutant cutaneous melanoma previously treated with or without a BRAF inhibitor. J Clin Oncol.

[116] Greger JG, Eastman SD, Zhang V, Bleam MR, Hughes AM, Smitheman KN, Dickerson SH, Laquerre SG, Liu L, Gilmer TM (2012). Combinations of BRAF, MEK, and PI3K/mTOR inhibitors overcome acquired resistance to the BRAF inhibitor GSK2118436 dabrafenib, mediated by NRAS or MEK mutations. Mol Cancer Ther.

[117] Johnson DB, Flaherty KT, Weber JS, Infante JR, Kim KB, Kefford RF, Hamid O, Schuchter L, Cebon J, Sharfman WH, McWilliams RR, Sznol M, Lawrence DP (2014). Combined BRAF (Dabrafenib) and MEK inhibition (Trametinib) in patients with BRAFV600-mutant melanoma experiencing progression with single-agent BRAF inhibitor. J Clin Oncol.

[118] Carlino MS, Todd JR, Gowrishankar K, Mijatov B, Pupo GM, Fung C, Snoyman S, Hersey P, Long GV, Kefford RF, Rizos H (2014). Differential activity of MEK and ERK inhibitors in BRAF inhibitor resistant melanoma. Mol Oncol.

[119] Morris EJ, Jha S, Restaino CR, Dayananth P, Zhu H, Cooper A, Carr D, Deng Y, Jin W, Black S, Long B, Liu J, Dinunzio E (2013). Discovery of a novel ERK inhibitor with activity in models of acquired resistance to BRAF and MEK inhibitors. Cancer Discov.

[120] Shin SY, Rath O, Choo SM, Fee F, McFerran B, Kolch W, Cho KH (2009). Positive- and negative-feedback regulations coordinate the dynamic behavior of the Ras-Raf-MEK-ERK signal transduction pathway. J Cell Sci.

[121] Sanchez-Hernandez I, Baquero P, Calleros L, Chiloeches A (2012). Dual inhibition of (V600E)BRAF and the PI3K/AKT/mTOR pathway cooperates to induce apoptosis in melanoma cells through a MEK-independent mechanism. Cancer Lett.

[122] Britten CD (2013). PI3K and MEK inhibitor combinations: examining the evidence in selected tumor types. Cancer Chemother Pharmacol.

[123] Das Thakur M, Salangsang F, Landman AS, Sellers WR, Pryer NK, Levesque MP, Dummer R, McMahon M, Stuart DD (2013). Modelling vemurafenib resistance in melanoma reveals a strategy to forestall drug resistance. Nature.

[124] Basile KJ, Le K, Hartsough EJ, Aplin AE (2014). Inhibition of mutant BRAF splice variant signaling by next-generation, selective RAF inhibitors. Pigment Cell Melanoma Res.

[125] Seghers AC, Wilgenhof S, Lebbe C, Neyns B (2012). Successful rechallenge in two patients with BRAF-V600-mutant melanoma who experienced previous progression during treatment with a selective BRAF inhibitor. Melanoma Res.

[126] Abdel-Wahab O, Klimek VM, Gaskell AA, Viale A, Cheng D, Kim E, Rampal R, Bluth M, Harding JJ, Callahan MK, Merghoub T, Berger MF, Solit DB (2014). Efficacy of intermittent combined RAF and MEK inhibition in a patient with concurrent BRAF- and NRAS-mutant malignancies. Cancer Discov.

[127] Puzanov I, Callahan MK, Linette GP, Patel SP, Luke JJ, Sosman JA, Wolchok JD, Hamid O, Minor DR, Orford KW, Hug BA, Ma B, Matthys GM (2014). Phase 1 study of the BRAF inhibitor dabrafenib (D) with or without the MEK inhibitor trametinib (T) in combination with ipilimumab (Ipi) for V600E/K mutation–positive unresectable or metastatic melanoma (MM). J Clin Oncol.

[128] Liu L, Mayes PA, Eastman S, Shi H, Yadavilli S, Zhang T, Yang J, Seestaller-Wehr L, Zhang SY, Hopson C, Tsvetkov L, Jing J, Zhang S (2015). The BRAF and MEK inhibitors dabrafenib and trametinib: effects on immune function and in combination with immunomodulatory antibodies targeting PD-1, PD-L1, and CTLA-4. Clin Cancer Res.

[129] Knight DA, Ngiow SF, Li M, Parmenter T, Mok S, Cass A, Haynes NM, Kinross K, Yagita H, Koya RC, Graeber TG, Ribas A, McArthur GA (2016). Host immunity contributes to the anti-melanoma activity of BRAF inhibitors. J Clin Invest.

[130] Ngiow SF, Knight DA, Ribas A, McArthur GA, Smyth MJ (2013). BRAF-targeted therapy and immune responses to melanoma. Oncoimmunology.

[131] Lenschow DJ, Walunas TL, Bluestone JA (1996). CD28/B7 system of T cell costimulation. Annu Rev Immunol.

[132] Lee PP, Yee C, Savage PA, Fong L, Brockstedt D, Weber JS, Johnson D, Swetter S, Thompson J, Greenberg PD, Roederer M, Davis MM (1999). Characterization of circulating T cells specific for tumor-associated antigens in melanoma patients. Nature Med.

[133] Ott PA, Hodi FS, Robert C (2013). CTLA-4 and PD-1/PD-L1 blockade: new immunotherapeutic modalities with durable clinical benefit in melanoma patients. Clin Cancer Res.

[134] Linnemann C, van Buuren MM, Bies L, Verdegaal EM, Schotte R, Calis JJ, Behjati S, Velds A, Hilkmann H, Atmioui DE, Visser M, Stratton MR, Haanen JB (2015). High-throughput epitope discovery reveals frequent recognition of neo-antigens by CD4+ T cells in human melanoma. Nature Med.

[135] Linette GP, Stadtmauer EA, Maus MV, Rapoport AP, Levine BL, Emery L, Litzky L, Bagg A, Carreno BM, Cimino PJ, Binder-Scholl GK, Smethurst DP, Gerry AB (2013). Cardiovascular toxicity and titin cross-reactivity of affinity-enhanced T cells in myeloma and melanoma. Blood.

[136] Hodi FS, O'Day SJ, McDermott DF, Weber RW, Sosman JA, Haanen JB, Gonzalez R, Robert C, Schadendorf D, Hassel JC, Akerley W, van den Eertwegh AJ, Lutzky J (2010). Improved survival with ipilimumab in patients with metastatic melanoma. N Engl J Med.

[137] Schartz NE, Farges C, Madelaine I, Bruzzoni H, Calvo F, Hoos A, Lebbe C (2010). Complete regression of a previously untreated melanoma brain metastasis with ipilimumab. Melanoma Res.

[138] Leach DR, Krummel MF, Allison JP (1996). Enhancement of antitumor immunity by CTLA-4 blockade. Science.

[139] McCoy KD, Le Gros G (1999). The role of CTLA-4 in the regulation of T cell immune responses. Immunol Cell Biol.

[140] Qureshi OS, Zheng Y, Nakamura K, Attridge K, Manzotti C, Schmidt EM, Baker J, Jeffery LE, Kaur S, Briggs Z, Hou TZ, Futter CE, Anderson G (2011). Trans-endocytosis of CD80 and CD86: a molecular basis for the cell-extrinsic function of CTLA-4. Science.

[141] Pentcheva-Hoang T, Simpson TR, Montalvo-Ortiz W, Allison JP (2014). Cytotoxic T lymphocyte antigen-4 blockade enhances antitumor immunity by stimulating melanoma-specific T-cell motility. Cancer Immunol Res.

[142] Robert C, Thomas L, Bondarenko I, O'Day S, Weber J, Garbe C, Lebbe C, Baurain JF, Testori A, Grob JJ, Davidson N, Richards J, Maio M (2011). Ipilimumab plus dacarbazine for previously untreated metastatic melanoma. N Engl J Med.

[143] Prieto PA, Yang JC, Sherry RM, Hughes MS, Kammula US, White DE, Levy CL, Rosenberg SA, Phan GQ (2012). CTLA-4 blockade with ipilimumab: long-term follow-up of 177 patients with metastatic melanoma. Clin Cancer Res.

[144] Di Giacomo AM, Ascierto PA, Queirolo P, Pilla L, Ridolfi R, Santinami M, Testori A, Simeone E, Guidoboni M, Maurichi A, Orgiano L, Spadola G, Del Vecchio M (2015). Three-year follow-up of advanced melanoma patients who received ipilimumab plus fotemustine in the Italian Network for Tumor Biotherapy (NIBIT)-M1 phase II study. Ann Oncol.

[145] Margolin K, Ernstoff MS, Hamid O, Lawrence D, McDermott D, Puzanov I, Wolchok JD, Clark JI, Sznol M, Logan TF, Richards J, Michener T, Balogh A (2012). Ipilimumab in patients with melanoma and brain metastases: an open-label, phase 2 trial. Lancet Oncol.

[146] Hodi FS, Mihm MC, Soiffer RJ, Haluska FG, Butler M, Seiden MV, Davis T, Henry-Spires R, MacRae S, Willman A, Padera R, Jaklitsch MT, Shankar S (2003). Biologic activity of cytotoxic T lymphocyte-associated antigen 4 antibody blockade in previously vaccinated metastatic melanoma and ovarian carcinoma patients. Proc Natl Acad Sci USA.

[147] Hodi FS, Butler M, Oble DA, Seiden MV, Haluska FG, Kruse A, Macrae S, Nelson M, Canning C, Lowy I, Korman A, Lautz D, Russell S (2008). Immunologic and clinical effects of antibody blockade of cytotoxic T lymphocyte-associated antigen 4 in previously vaccinated cancer patients. Proc Natl Acad Sci USA.

[148] Tarhini A (2013). Immune-mediated adverse events associated with ipilimumab ctla-4 blockade therapy: the underlying mechanisms and clinical management. Scientifica.

[149] Carreno BM, Collins M (2002). The B7 family of ligands and its receptors: new pathways for costimulation and inhibition of immune responses. Ann Rev Immunol.

[150] Taube JM, Anders RA, Young GD, Xu H, Sharma R, McMiller TL, Chen S, Klein AP, Pardoll DM, Topalian SL, Chen L (2012). Colocalization of inflammatory response with B7-h1 expression in human melanocytic lesions supports an adaptive resistance mechanism of immune escape. Sci Transl Med.

[151] Wang W, Lau R, Yu D, Zhu W, Korman A, Weber J (2009). PD1 blockade reverses the suppression of melanoma antigen-specific CTL by CD4+ CD25(Hi) regulatory T cells. Int Immunol.

[152] Madore J, Vilain RE, Menzies AM (2015). PD-L1 expression in melanoma shows marked heterogeneity within and between patients: implications for anti-PD-1 and PD-L1 clinical trials. Pigment Cell Melanoma Res.

[153] Robert C, Long GV, Brady B, Dutriaux C, Maio M, Mortier L, Hassel JC, Rutkowski P, McNeil C, Kalinka-Warzocha E, Savage KJ, Hernberg MM, Lebbe C (2015). Nivolumab in previously untreated melanoma without BRAF mutation. N Engl J Med.

[154] Topalian SL, Sznol M, McDermott DF, Kluger HM, Carvajal RD, Sharfman WH, Brahmer JR, Lawrence DP, Atkins MB, Powderly JD, Leming PD, Lipson EJ, Puzanov I (2014). Survival, durable tumor remission, and long-term safety in patients with advanced melanoma receiving nivolumab. J Clin Oncol.

[155] Sznol M, Kluger HM, Hodi FS, McDermott DF, Carvajal RD, Lawrence DP, Topalian SL, Atkins MB, Powderly JD, Sharfman WH, Puzanov I, Smith DC, Wigginton JM (2013). Survival and long-term follow-up of safety and response in patients (pts) with advanced melanoma (MEL) in a phase I trial of nivolumab. J Clin Oncol.

[156] Hamid O, Robert C, Daud A, Hodi FS, Hwu WJ, Kefford R, Wolchok JD, Hersey P, Joseph RW, Weber JS, Dronca R, Gangadhar TC, Patnaik A (2013). Safety and tumor responses with lambrolizumab (anti-PD-1) in melanoma. N Engl J Med.

[157] Park JJ, Parakh S, Mendis S, Rai R, Lo S, Haydon A, Andrews MC, Cebon J, Guminski A, Kefford R, Long GV, Menzies AM, Klein O (2016). Efficacy of anti-PD-1 therapy in patients with melanoma brain metastases. Ann Oncol.

[158] Brunot A, Jeudy G, Tas M, Guillot B, Kramkimel N, Mortier L, Mansard S, Lebbe C, Blom A, Le Corre Y, Montaudie H, Prey S, Campillo-Giminez B (2016). Anti-PD-1 tolerance after severe toxicity with ipilimumab therapy in metastatic melanoma patients. J Clin Oncol.

[159] Page DB, Postow MA, Callahan MK, Allison JP, Wolchok JD (2014). Immune modulation in cancer with antibodies. Annu Rev Med.

[160] Massi D, Brusa D, Merelli B, Ciano M, Audrito V, Serra S, Buonincontri R, Baroni G, Nassini R, Minocci D, Cattaneo L, Tamborini E, Carobbio A (2014). PD-L1 marks a subset of melanomas with a shorter overall survival and distinct genetic and morphological characteristics. Ann Oncol.

[161] Topalian SL, Hodi FS, Brahmer JR, Gettinger SN, Smith DC, McDermott DF, Powderly JD, Carvajal RD, Sosman JA, Atkins MB, Leming PD, Spigel DR, Antonia SJ (2012). Safety, activity, and immune correlates of anti-PD-1 antibody in cancer. N Engl J Med.

[162] Brahmer JR, Tykodi SS, Chow LQ, Hwu WJ, Topalian SL, Hwu P, Drake CG, Camacho LH, Kauh J, Odunsi K, Pitot HC, Hamid O, Bhatia S (2012). Safety and activity of anti-PD-L1 antibody in patients with advanced cancer. N Engl J Med.

[163] Wolchok JD, Kluger H, Callahan MK, Postow MA, Rizvi NA, Lesokhin AM, Segal NH, Ariyan CE, Gordon RA, Reed K, Burke MM, Caldwell A, Kronenberg SA (2013). Nivolumab plus ipilimumab in advanced melanoma. N Engl J Med.

[164] Postow MA, Chesney J, Pavlick AC, Robert C, Grossmann K, McDermott D, Linette GP, Meyer N, Giguere JK, Agarwala SS, Shaheen M, Ernstoff MS, Minor D (2015). Nivolumab and ipilimumab versus ipilimumab in untreated melanoma. N Engl J Med.

[165] Curran MA, Montalvo W, Yagita H, Allison JP (2010). PD-1 and CTLA-4 combination blockade expands infiltrating T cells and reduces regulatory T and myeloid cells within B16 melanoma tumors. Proc Natl Acad Sci USA.

[166] Atkins MB, Lotze MT, Dutcher JP, Fisher RI, Weiss G, Margolin K, Abrams J, Sznol M, Parkinson D, Hawkins M, Paradise C, Kunkel L, Rosenberg SA (1999). High-dose recombinant interleukin 2 therapy for patients with metastatic melanoma: analysis of 270 patients treated between 1985 and 1993. J Clin Oncol.

[167] Agarwala SS (2009). Current systemic therapy for metastatic melanoma. Expert Rev Anticancer Ther.

[168] Sosman JA, Carrillo C, Urba WJ, Flaherty L, Atkins MB, Clark JI, Dutcher J, Margolin KA, Mier J, Gollob J, Kirkwood JM, Panka DJ, Crosby NA (2008). Three phase II cytokine working group trials of gp100 (210M) peptide plus high-dose interleukin-2 in patients with HLA-A2-positive advanced melanoma. J Clin Oncol.

[169] Rosenberg SA, Dudley ME (2009). Adoptive cell therapy for the treatment of patients with metastatic melanoma. Curr Opin Immunol.

[170] Dudley ME, Yang JC, Sherry R, Hughes MS, Royal R, Kammula U, Robbins PF, Huang J, Citrin DE, Leitman SF, Wunderlich J, Restifo NP, Thomasian A (2008). Adoptive cell therapy for patients with metastatic melanoma: evaluation of intensive myeloablative chemoradiation preparative regimens. J Clin Oncol.

[171] Knisely JP, Yu JB, Flanigan J, Sznol M, Kluger HM, Chiang VL (2012). Radiosurgery for melanoma brain metastases in the ipilimumab era and the possibility of longer survival. J Neurosurg.

[172] Mathew M, Tam M, Ott PA, Pavlick AC, Rush SC, Donahue BR, Golfinos JG, Parker EC, Huang PP, Narayana A (2013). Ipilimumab in melanoma with limited brain metastases treated with stereotactic radiosurgery. Melanoma Res.

[173] Formenti SC (2015). Combining radiation therapy with immunotherapy: clinical translation. J Transl Med.

[174] Dunn GP, Old LJ, Schreiber RD (2004). The three Es of cancer immunoediting. Annu Rev Immunol.

[175] Castelli C, Rivoltini L, Andreola G, Carrabba M, Renkvist N, Parmiani G (2000). T-cell recognition of melanoma-associated antigens. J Cell Physiol.

[176] Landsberg J, Kohlmeyer J, Renn M, Bald T, Rogava M, Cron M, Fatho M, Lennerz V, Wolfel T, Holzel M, Tuting T (2012). Melanomas resist T-cell therapy through inflammation-induced reversible dedifferentiation. Nature.

[177] Ribas A, Tumeh PC (2012). Cancer therapy: tumours switch to resist. Nature.

[178] Spranger S, Spaapen RM, Zha Y, Williams J, Meng Y, Ha TT, Gajewski TF (2013). Up-regulation of PD-L1, IDO, and T(regs) in the melanoma tumor microenvironment is driven by CD8(+) T cells. Sci Transl Med.

[179] Jazirehi AR, Kurdistani SK, Economou JS (2014). Histone deacetylase inhibitor sensitizes apoptosis-resistant melanomas to cytotoxic human T lymphocytes through regulation of TRAIL/DR5 pathway. J Immunol.

[180] Tse AK, Cao HH, Cheng CY, Kwan HY, Yu H, Fong WF, Yu ZL (2014). Indomethacin sensitizes TRAIL-resistant melanoma cells to TRAIL-induced apoptosis through ROS-mediated upregulation of death receptor 5 and downregulation of survivin. J Invest Dermatol.

[181] Restifo NP, Dudley ME, Rosenberg SA (2012). Adoptive immunotherapy for cancer: harnessing the T cell response. Nature Rev Immunol.

[182] Pardoll DM (2012). The blockade of immune checkpoints in cancer immunotherapy. Nature Rev Cancer.

[183] Del Campo AB, Kyte JA, Carretero J, Zinchencko S, Mendez R, Gonzalez-Aseguinolaza G, Ruiz-Cabello F, Aamdal S, Gaudernack G, Garrido F, Aptsiauri N (2014). Immune escape of cancer cells with beta2-microglobulin loss over the course of metastatic melanoma. Int J Cancer.

[184] Weber JS, Kudchadkar RR, Yu B, Gallenstein D, Horak CE, Inzunza HD, Zhao X, Martinez AJ, Wang W, Gibney G, Kroeger J, Eysmans C, Sarnaik AA (2013). Safety, efficacy, and biomarkers of nivolumab with vaccine in ipilimumab-refractory or -naive melanoma. J Clin Oncol.

[185] Robert C, Schadendorf D, Messina M, Hodi FS, O'Day S (2013). Efficacy and safety of retreatment with ipilimumab in patients with pretreated advanced melanoma who progressed after initially achieving disease control. Clin Cancer Res.

[186] Lipson EJ, Sharfman WH, Drake CG, Wollner I, Taube JM, Anders RA, Xu H, Yao S, Pons A, Chen L, Pardoll DM, Brahmer JR, Topalian SL (2013). Durable cancer regression off-treatment and effective reinduction therapy with an anti-PD-1 antibody. Clin Cancer Res.

[187] Ribas A, Hersey P, Middleton MR, Gogas H, Flaherty KT, Sondak VK, Kirkwood JM (2012). New challenges in endpoints for drug development in advanced melanoma. Clin Cancer Res.

